# Selenide-linked polydopamine-reinforced hybrid hydrogels with on-demand degradation and light-triggered nanozyme release for diabetic wound healing

**DOI:** 10.1186/s40824-023-00367-w

**Published:** 2023-05-18

**Authors:** Wenjing Li, Ying Bei, Xiangqiang Pan, Jian Zhu, Zhengbiao Zhang, Tinglin Zhang, Jieting Liu, Dan Wu, Meng Li, Yan Wu, Jie Gao

**Affiliations:** 1 Changhai Clinical Research Unit, Shanghai Changhai Hospital, Naval Medical University, Shanghai, 200433 China; 2 Jiangsu Key Laboratory of Advanced Functional Polymer Design and Application, Department of Polymer Science and Engineering, College of Chemistry, Chemical Engineering and Materials Science, Soochow University, Suzhou, 215123 China; 3 College of Life Science, Mudanjiang Medical University, Mudanjiang, 157011 China; 4 Department of Dermatology, Shanghai Ninth People?s Hospital, Shanghai Jiaotong University, Shanghai, 200010 China

**Keywords:** Diselenide, Hydrogels, Polydopamine, Prussian blue, Wound healing

## Abstract

**Background:**

Multifunctional hydrogels with controllable degradation and drug release have attracted extensive attention in diabetic wound healing. This study focused on the acceleration of diabetic wound healing with selenide-linked polydopamine-reinforced hybrid hydrogels with on-demand degradation and light-triggered nanozyme release.

**Methods:**

Herein, selenium-containing hybrid hydrogels, defined as DSeP@PB, were fabricated *via* the reinforcement of selenol-end capping polyethylene glycol (PEG) hydrogels by polydopamine nanoparticles (PDANPs) and Prussian blue nanozymes in a one-pot approach in the absence of any other chemical additive or organic solvent based on diselenide and selenide bonding-guided crosslinking, making them accessible for large-scale mass production.

**Results:**

Reinforcement by PDANPs greatly increases the mechanical properties of the hydrogels, realizing excellent injectability and flexible mechanical properties for DSeP@PB. Dynamic diselenide introduction endowed the hydrogels with on-demand degradation under reducing or oxidizing conditions and light-triggered nanozyme release. The bioactivity of Prussian blue nanozymes afforded the hydrogels with efficient antibacterial, ROS-scavenging and immunomodulatory effects, which protected cells from oxidative damage and reduced inflammation. Further animal studies indicated that DSeP@PB under red light irradiation showed the most efficient wound healing activity by stimulating angiogenesis and collagen deposition and inhibiting inflammation.

**Conclusion:**

The combined merits of DSeP@PB (on-demand degradation, light-triggered release, flexible mechanical robustness, antibacterial, ROS-scavenging and immunomodulatory capacities) enable its high potential as a new hydrogel dressing that can be harnessed for safe and efficient therapeutics for diabetic wound healing.

**Supplementary Information:**

The online version contains supplementary material available at 10.1186/s40824-023-00367-w.

## Introduction

Diabetic wounds are serious complications that lead to severe morbidity and mortality and may require limb amputation for treatment [1]. In diabetic wounds, inefficient wound healing is usually caused by reduced blood flow, delayed extracellular matrix turnover, decreased wound contraction, repeated infections and chronic inflammation [2, 3]. Hydrogels, which act as scaffold materials, have been extensively used in diabetic wounds owing to their unique merits, including high water content, soft texture, highly adjustable mechanical properties, good biocompatibility and easy functionalization. Hydrogels may absorb wound exudates, keep the wound environment moist, enable oxygen diffusion, and potentially have antibacterial effects because of their high water content, excellent hydrophilicity, and high porosity [4, 5]. However, the pathological process of diabetic wounds is complex and characterized by decreased proliferation, impaired angiogenesis, persistent infections, and chronic inflammation, and these processes often overlap and have a mutual influence on diabetic wound healing [6–8]. Although hydrogels do not shed many contaminants on wounds such as fibers, unlike the case with standard gauze and cotton wool dressings, the residue of hydrogels may hinder wound healing and increase wound infection and inflammation, making it necessary to design hydrogels with on-demand degradation [9–13]. Furthermore, due to the long and complicated pathological process of diabetic wounds, stimuli-responsive drug delivery approaches provide considerable promise for promoting wound healing [14–16]. Therefore, functional components of hydrogels should undergo on-demand degradation and allow for controllable drug release, thus offering safe dressing exchange and controllable delivery of drugs, which is a key strategy for diabetic wound healing.

Hydrogels with controllable drug release and on-demand degradation can be prepared by rationally designing synthetic raw materials and cross-linking the functional components and chemical structures [17]. The stimuli-responsive characteristics and highly efficient drug-carrying capacity of selenium-containing polymers make them excellent options for smart drug delivery systems [18–20]. Our prior research focused on the development of selenium-containing polymers with a variety of topological characteristics, including both main-chain and hyperbranched selenium-containing polymers, along with side-chain selenium-containing block copolymers [21, 22]. We have demonstrated that upon the addition of H_2_O_2_ or dithiothreitol (DTT), poly(2-ethyl-2-oxazoline) (PEtOx) diselenide-containing hydrogels gradually disassemble as a result of Se-Se bond structural dissociation [13]. In another study, we showed that increasing the temperature can accelerate the dynamic exchange process of Se-Se bonds [23]. These favorable properties make diselenide-containing hydrogels suitable for on-demand degradation and controllable drug release as diabetic wound dressings. However, the poor mechanical properties of diselenide-containing hydrogels limit their biomedical applications due to the sensitive diselenide bond, which cannot maintain the rigid structure of hydrogels [24].

Hybrid hydrogels that integrate nanoparticles into their hydrogel networks through chemical interactions can be tailored to promote the mechanical properties of hydrogels [25]. Polydopamine nanoparticles (PDANPs), composed of the major component of melanin, possess distinct properties, such as good biocompatibility and mechanical properties [26, 27]. Importantly, PDANPs could be used as crosslinking agents to crosslink thiol-terminated chemicals through nucleophilic reactions [28]. Owing to the chemical similarity between thiol and selenol groups, we hypothesize that selenide-linked hydrogels could also be cross-linked with PDANPs, resulting in the enhanced mechanical properties of PDANP hybrid selenide-linked hydrogels. Furthermore, the chemical crosslinking and physical bonding between PDA and soft tissue endow PDA hydrogels with exceptional tissue adhesion capabilities [29], and the introduction of catechol to hydrogels through PDA could also improve the antioxidant effects of hydrogels [25, 30]. Therefore, the incorporation of PDANPs into selenide-linked hydrogels is an attractive design approach that increases the mechanical, adhesive, antibacterial and antioxidant properties of hydrogels [31, 32].

Nanozymes are functional components that exert multiple functionalities, such as antioxidant, antibacterial, and photothermal properties [33]. Prussian blue (PB) nanozymes, cyano-bridged coordination nanoparticles, have unique magnetic, photomagnetic and electrochemical properties [34]. Importantly, PB nanozymes possess peroxidase, superoxide dismutase, and catalase (CAT) activities [35]. When exposed to 730 nm irradiation, PB nanozymes exhibit an enhanced photothermal conversion efficiency, causing them to produce antioxidant activity akin to that of an enzyme, as well as photothermal activity effective against bacteria [36]. Therefore, the incorporation of PB nanozymes could afford hydrogels with efficient anti-infection, ROS-scavenging and immunomodulatory effects.

**Scheme 1:**
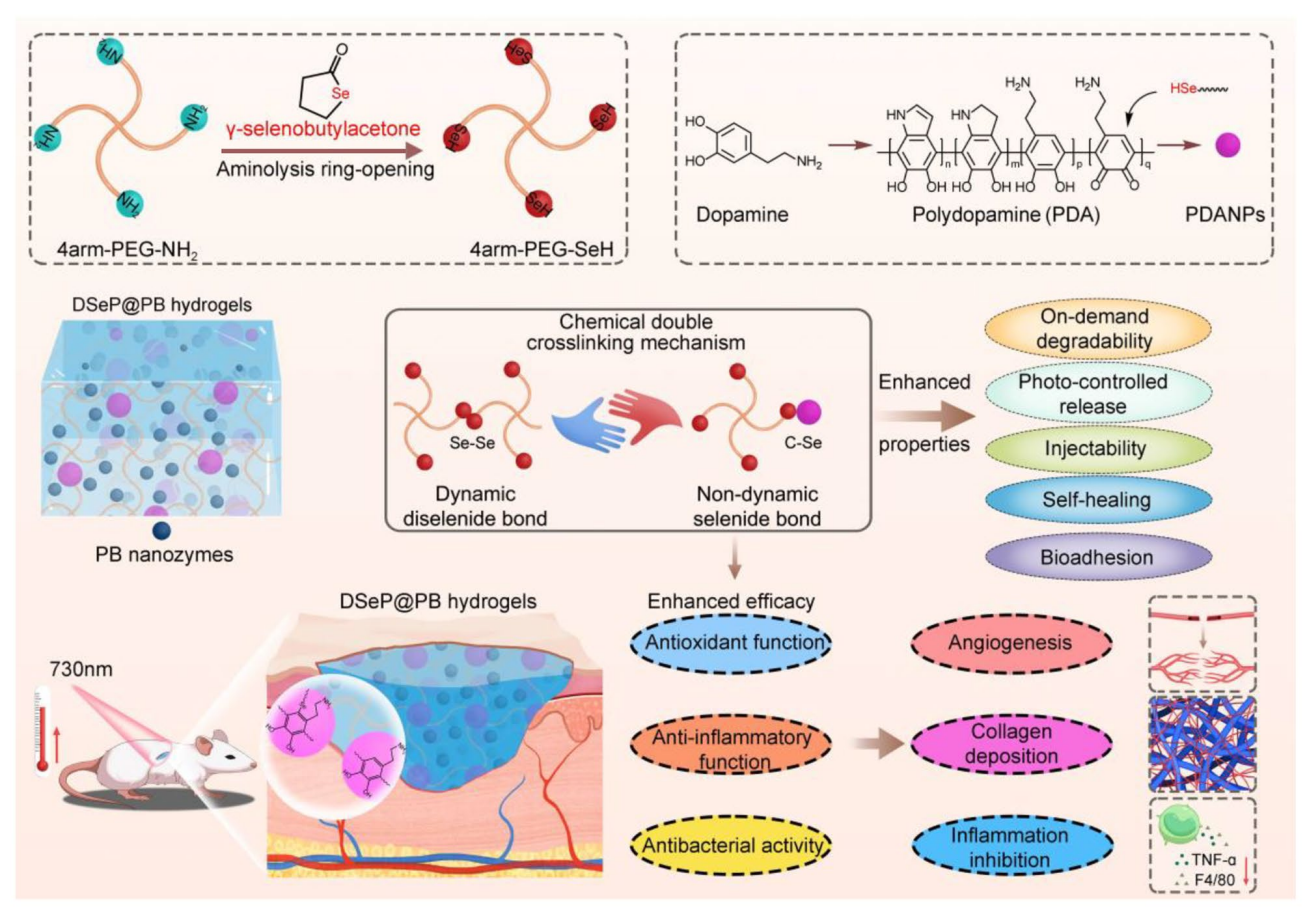
The development and characteristics of DSeP@PB for the treatment of diabetic wounds. The hydrogels were fabricated *via* reinforcement of diselenide-containing polyethylene glycol (PEG) hydrogels synthesized by ring-opening of γ-selenobutylacetone by polydopamine nanoparticles (PDANPs) and Prussian blue nanozymes in a one-pot low-cost green approach. Owing to the chemical double cross-linking mechanisms (diselenide and selenide bonds), DSeP@PB possesses distinct properties, such as on-demand degradability, photocontrolled release, injectability, self-healing and bioadhesion, realizing enhanced efficacy (antioxidant functions, anti-inflammatory functions and antibacterial activity) to promote diabetic wound healing by increased angiogenesis, collagen deposition, and inflammation inhibition

Here, a new selenide-linked PDA-reinforced hybrid hydrogel was developed as a dressing material with controllable degradation and drug release to promote rapid diabetic wound healing. The hybrid hydrogel, defined as DSeP@PB, is composed of dynamic diselenide-containing hydrogels reinforced by crosslinking with PDANPs and PB nanozyme incorporation, thus realizing both controllable degradation and drug release. Additionally, the cross-linking of PDANPs improved the hydrogel strength after injection into the wound sites. Thus, the chemical double-crosslinking mechanism, including the dynamic diselenide bond and nondynamic selenide, provides a general strategy to realize controllable degradation and drug release, injectability, self-healing and bioadhesion. More importantly, incorporation of PB nanozymes afforded hydrogels with anti-infection, ROS-scavenging and immunomodulatory effects (Scheme 1). Compared with previously reported hydrogels, DSeP@PB exhibits controllable degradation and drug release with excellent injectability, mechanical strength and bioactivity without costly and complicated preparation procedures, demonstrating a safe and efficient therapeutic for diabetic wound dressing.

## Materials and methods

### Polydopamine nanoparticle (PDANP) synthesis

PDANPs were prepared using the same procedure as reported before [25]. Additional information is provided in the Supplementary Materials and Methods.

### Preparation of PB nanozymes

PB nanozymes were synthesized *via* a simple hydrothermal method [37]. Additional data are provided in the Supplementary Materials and Methods.

### Synthesis of 4-arm-PEG-SeH and hydrogels

The preparation of 4-arm polyethylene glycol with selenol (4-arm-PEG-SeH) followed the protocol outlined in our earlier research [13]. The hydrogel was prepared by mixing PDANPs with 4-armed PEG-SeH and PBNPs in an aqueous solution. Additional information is provided in the Supplementary Materials and Methods.

### NMR, FTIR, XPS and FESEM analysis

On a Bruker Avance 300 system (Bruker, CA, USA), ^1^ H NMR spectra were determined in D_2_O at 300 MHz. For Fourier transform infrared spectroscopy (FTIR), 4-arm-PEG-NH_2_ and freeze-dried hydrogels were ground to powder and analyzed using KBr pellets on a Bruker TENSOR 27 FTIR system (Bruker Daltonics Inc., CA, USA). The element composition and valency changes in the polymers were measured *via* X-ray photoelectron spectroscopy (XPS) (Thermo Fisher Scientific, ESCALAB 250 XI, Al KR source, MA, USA). The morphology of the hydrogels was examined on a field emission scanning electron microscope (FESEM, Hitachi S-4700, Tokyo, Japan, 15 kV). Additional data are rarely provided in the Supplementary Materials and Methods.

### Rheological measurements

A rheometer (RS 6000, HAAKE, Berlin, Germany) was employed to determine the rheological characteristics of the hydrogels. Supplementary Materials and Methods provide further information.

### Swelling behavior of hydrogels

After the hydrogels were dried to form flakes (5 mm × 5 mm × 2 mm) and subsequently freeze-dried, they were collected in a vial, weighed and dipped in 10 mL of PBS (10 mM, pH 7.4) at 37 °C. The hydrogels were removed at regular intervals, wiped using filter paper, and then weighed. At a variety of time points, the mass of the hydrogels, denoted by M_t_, was measured and recorded. The mass of the samples at a fixed time interval was weighed to calculate the swelling ratio using the formula below:

Swelling ratio = (M_t_ – M_0_)/M_0_ × 100% (where M_t_ is the mass of hydrogel samples at a fixed time point, and M_0_ is the mass of the original hydrogel samples).

### Analysis of tissue adhesion strength

The strength of hydrogel adhesion was determined by performing a tensile lap-shear test at 37 °C on a universal testing machine (Tinius Olsen model H10KS, Tinius Olsen, USA), as specified by American Society for Testing and Materials (ASTM) standard F2255-05. Additional data are displayed in the Supplementary Materials and Methods.

### Photothermal effects of hydrogels

Hydrogels (2 g) were collected in a 10-mL Eppendorf tube, after which they were subjected to 26 min of irradiation with 730-nm red light. An infrared thermal imaging camera (HT-18, Xinste, Hubei, China) was used to record the infrared pictures, and the temperature was recorded every 2 min. By analyzing the heating and cooling curves, the hydrogels’ resistance to heat was validated. The hydrogels were initially subjected to 730-nm red light (1 W·cm^-2^) and subsequently moved to the dark after 5 min. These cycles were repeated several times.

### Release behavior of PB nanozymes from hydrogels

Hydrogels (2 g) in PBS (pH 7.4) were irradiated with a 730-nm red light at a power density of 1 W·cm^-2^, and 200 µL of suspension was removed at specified time periods. Next, the solution was seared on a Muffle stove (300 °C, 4 h) to remove organic matter and dissolved in 1% dilute nitric acid (5 mL). Moreover, inductively coupled plasma–mass spectrometry (ICP‒MS, Bruker ICPOES 5110, Bruker Daltonics Inc., CA, USA) was used to measure the concentration of Fe^3+^ in the sediment.

### Hydrogen peroxide degradation assay

H_2_O_2_ was combined with PB nanozymes at different doses, and then the proportion of H_2_O_2_ that remained after this process was determined using a xylenol orange assay following the guidelines provided by the manufacturer (Quantitative Peroxide Assay Kit, Beyotime, Shanghai, China). Additional data are presented in the Supplementary Materials and Methods.

### Superoxide scavenging activity

The superoxide-scavenging activity of PB nanozymes was analyzed using a superoxide dismutase (SOD) assay kit (Nanjing Jiancheng Bioengineering Institute, Nanjing, China) as described in our previous study [33]. Supplementary Materials and Methods provide more information.

### Biocompatibility assay

The biocompatibility of hydrogels and PB nanozymes was evaluated *via* calcein acetoxymethyl ester (calcein-AM) and propidium iodide (PI) double-staining and methylthiazoletetrazolium (MTT) assays. First, double labeling with calcein-AM and PI, as reported earlier, was performed to determine cell viability [38]. NIH-3T3 cells were treated with hydrogels and PB nanozymes before incubation at 37 °C for 24 h (all DSeP@PB and PB nanozymes contained 100 µg·mL^-1^ PB nanozymes). Samples in the groups treated with red light (RL) were irradiated with RL (730 nm, 1 W/cm^2^, 15 min, VCLHLGD0025017, Blueprint, Beijing, China). NIH-3T3 cells were stained for 20 min with red (PI) and green (calcein-AM) fluorescent dyes at 37 °C and imaged under a live cell imaging system (DMI6000B; Feica, Wetzlar, Germany). Second, cell viability was evaluated *via* the MTT assay. Additional information is provided in the Supplementary Materials and Methods.

### Generation of intracellular ROS and cytotoxicity assay

To examine intracellular reactive oxidative species (ROS) formation, the ROS-sensitive fluorescent probe dichlorodihydrofluorescein diacetate assay (DCFH-DA) was used as described previously [34]. Subconfluent NIH-3T3 cells were cocultured with 1 mL medium containing hydrogels and PB nanozymes (0.1 mL, DSeP@PB and PB nanozymes containing 100 µg·mL^-1^ PB nanozymes) with or without H_2_O_2_ (100 µM). Red light (1 W/cm^2^, 15 min) was used to illuminate the groups that were given the RL treatment. Following treatment for 24 h, the cells were rinsed and then placed in an incubator containing 10 µM DCFH-DA for 30 min. In addition, an intracellular fluorescent signal was observed with the aid of a live cell imaging system (DMI6000B; Feica, Wetzlar, Germany). To assess whether hydrogels and PB nanozymes have a protective impact on cells, a 96-well plate was used to cultivate NIH-3T3 cells, which were then subjected to 1 mL medium containing hydrogels and PB nanozymes (0.1 mL, DSeP@PB and PB nanozymes containing 100 µg·mL^-1^ PB nanozymes) in the presence of 100 µM H_2_O_2_. The cells were incubated for 24 h before being rinsed and subjected to the MTT assay to determine their viability.

### Hydrogels’ antibacterial characteristics *in vitro*

Hydrogels and PB nanozymes (0.3 mL, DSeP@PB and PB nanozyme groups containing 100 µg·mL^-1^ of PB) were introduced into 1 mL of bacterial solution (*S. aureus* and *E. coli*, 1 × 10^7^ CFU·mL^-1^), and the mixture was either subjected to light (730 nm, 1 W/cm^2^) or placed in darkness. Subsequently, the solution was diluted 1000 times, and 40 µL of the solution was extracted before being placed onto a culture plate. After 16 h of culture, images were captured and analyzed.

### Animal studies

Male Institute of Cancer Research (ICR) mice (weight, 25–30 g; age, 6–8 weeks) were provided by Beijing Vital River Laboratory Animal Technology Co., Ltd. (Beijing, China). All animal experiments were approved by and conducted following the guidelines of the Ethics Committee on Animals of Mudanjiang Medical University (Mudanjiang, China) (approval number: 20210810-02). Animal experiments were performed in a specific pathogen-free laboratory. Mice were housed in an environment with a relative humidity of 50–60%, a temperature of 20–24 °C, and a 12-h dark/light cycle. Finally, they were euthanized with carbon dioxide.

### Biological safety study

After randomly dividing the ICR mice, they were intradermally injected with 200 µL of saline, PB nanozymes, DSeP and DSeP@PB (DSeP@PB and PB nanozyme groups contained 100 µg·mL^-1^ of PB). RL (1 W/cm^2^, 15 min) was used to irradiate the RL treatment groups. Mice were weighed daily for 21 days throughout the treatment period. Serum was extracted by centrifuging the blood at 3000 × *g* for 15 min from blood extracted from the mice’s orbital vein on day 21. Subsequently, serum biochemical indicators were measured and included uric acid (UA), urea, aspartate aminotransferase (AST), and alanine aminotransferase (ALT). Mice from each group had their primary organs, including the kidney, spleen, lungs, liver, and heart, removed and fixed for 24 h in 4% paraformaldehyde (PFA), followed by paraffin embedding and staining with hematoxylin and eosin (H&E).

### *In vivo* antibacterial evaluation

After two days of acclimation, eight groups (three mice each) were established at random: control, light, DSeP, DSeP + RL, PB, PB + RL, DSeP@PB and DSeP@PB + RL groups. Each mouse had a wound made on its back with a diameter of 15 mm. After the injection of 0.1 mL of bacterial solution (10^7^ CFU·mL^-1^), mice in various groups received different care for their wounds. Next, the wounds were covered with 0.1 mL PBS in the control and light groups. In the DSeP and DSeP + RL groups, the wounds were covered with 0.1 mL of DSeP. In the PB and PB + RL groups, the wounds were covered with 0.1 mL of PB (100 µg·mL^-1^). In the DSeP@PB and DSeP@PB + RL groups, the wounds were covered with 0.1 mL DSeP@PB (containing 100 µg·mL^-1^ PB nanozymes). Every day, the RL groups were subjected to RL (730 nm, 1 W·cm^-2^, 15 min). On day 3, tissue fluid smeared by a sterile cotton stick from the wound site was mixed with 1 mL of bacterial medium. Thereafter, 40 µL of the solution was extracted and spread on a plate. After 16 h of culture, images were captured and analyzed.

### *In vivo* diabetic wound healing evaluation

To establish type 1 diabetes in male ICR mice, the mice were fasted overnight before having their abdominal cavity injected with 100 mg/kg of streptozotocin (STZ). This was followed by an injection of 50 mg/kg once daily for two days. One week later, blood glucose levels were monitored, and diabetes status was determined based on whether blood glucose levels were higher than 16.7 mM. Each mouse had two circular, full-thickness cutaneous lesions (diameter = 6 mm) made on its back. Seven groups of mice were established at random and treated with 200 µL of DSeP hydrogels, PB nanozymes, DSeP@PB or PBS (control). Mice in the DSeP + RL, PB + RL and DSeP@PB + RL groups were illuminated with 730-nm RL for 15 min with a power density of 1 W/cm^2^ (the DSeP@PB and PB nanozyme groups contained 100 µg·mL^-1^ of PB). On days 0, 3, 7, and 14, digital images of the wounds were captured using a digital camera to track their progress as they healed. The following formula was used to determine the wound healing rate: wound healing rate = (wound area at day 0 − wound area at a certain day)/wound area at day 0.

A histological investigation was performed using H&E and Masson’s trichrome stains, as per the manufacturer’s instructions. A Nikon FHEPSE 80i microscope (Nikon, Japan) was employed to examine the slides. To assess blood vessel formation together with anti-inflammatory effects during wound healing, immunostaining for α-SMA, CD31, F4/80 and TNF-α was performed. Additional information is provided in the Supplementary Materials and Methods.

### RNA-seq and data analysis

After treatment for 2 weeks, wound tissues were sectioned into smaller pieces and then immersed in RNAlater solution. Samples of RNA were analyzed with an Agilent 2100 bioanalyzer and a NanoDrop (Thermo Fisher Scientific, MA, USA) to determine their quality and quantity. The BGISEQ-500 platform was utilized for the construction and sequencing of RNA-seq libraries. The bioinformatics data were analyzed using the Majorbio Cloud Platform (Majorbio Biopharm Biotechnology, China). Sequencing quality was assessed using Fastx-Toolkit v.0.0.14. (http://hannonlab.cshl.edu/fastx_toolkit/) was used as a reference genome to map all reads. Gene abundance was quantified using RSEM v.1.3.1 (http://deweylab.biostat.wisc.edu/rsem). Differential gene expression was analyzed using DESeq2 v.1.24.0 (http://bioconductor.org/packages/stats/bioc/DESeq2/) (cutoff: fold change ≥ 2 and *p* value < 0.05). Heatmaps were generated, and hierarchical clustering was performed with Perseus v.1.6.1.1 (http://www.coxdocs.org/doku.php?id=perseus:start). KEGG functional enrichment analysis was performed with KOBAS v.2.1.1 (http://kobas.cbi.pku.edu.cn/download.php). In addition, the Search Tool for the Retrieval of Interacting Genes/Proteins (STRING) algorithm (http://www.string-db.org/) was used to excavate information on protein‒protein interactions.

### Statistical analysis

The data are presented as the mean ± standard deviation (SD). When comparing the means of two different groups, unpaired Student’s t test was utilized. Comparison of the means of three or more groups was carried out using one-way analysis of variance (ANOVA) with the Newman–Keuls test. (*, *P* < 0.05; **, *P* < 0.01; ***, *P* < 0.001; ****, *P* < 0.0001; n.s., not significant [*P* > 0.05]).

## Results and discussion

### Synthesis and characterization of polymers and hydrogels

Scheme 1 shows a schematic of the fabrication process for obtaining different hydrogels. First, 4-arm-PEG-SeH (4-arm polyethylene glycol (PEG) with selenol) was synthesized by linking ring-opening γ-selenobutylacetone to 4-arm-PEG-NH_2_. PEG-Se_2_ (diselenide-containing polyethylene glycol hydrogels) was then produced by oxidizing 4-arm-PEG-SeH. DSeP (PEG-Se_2_ loaded with PDANPs) and DSeP@PB (PEG-Se_2_ loaded with PDANPs and PB nanozymes) were produced by mixing 4-arm-PEG-SeH and PDANPs or 4-arm-PEG-SeH, PDANPs and PB nanozymes, respectively.

**Fig. 1:**
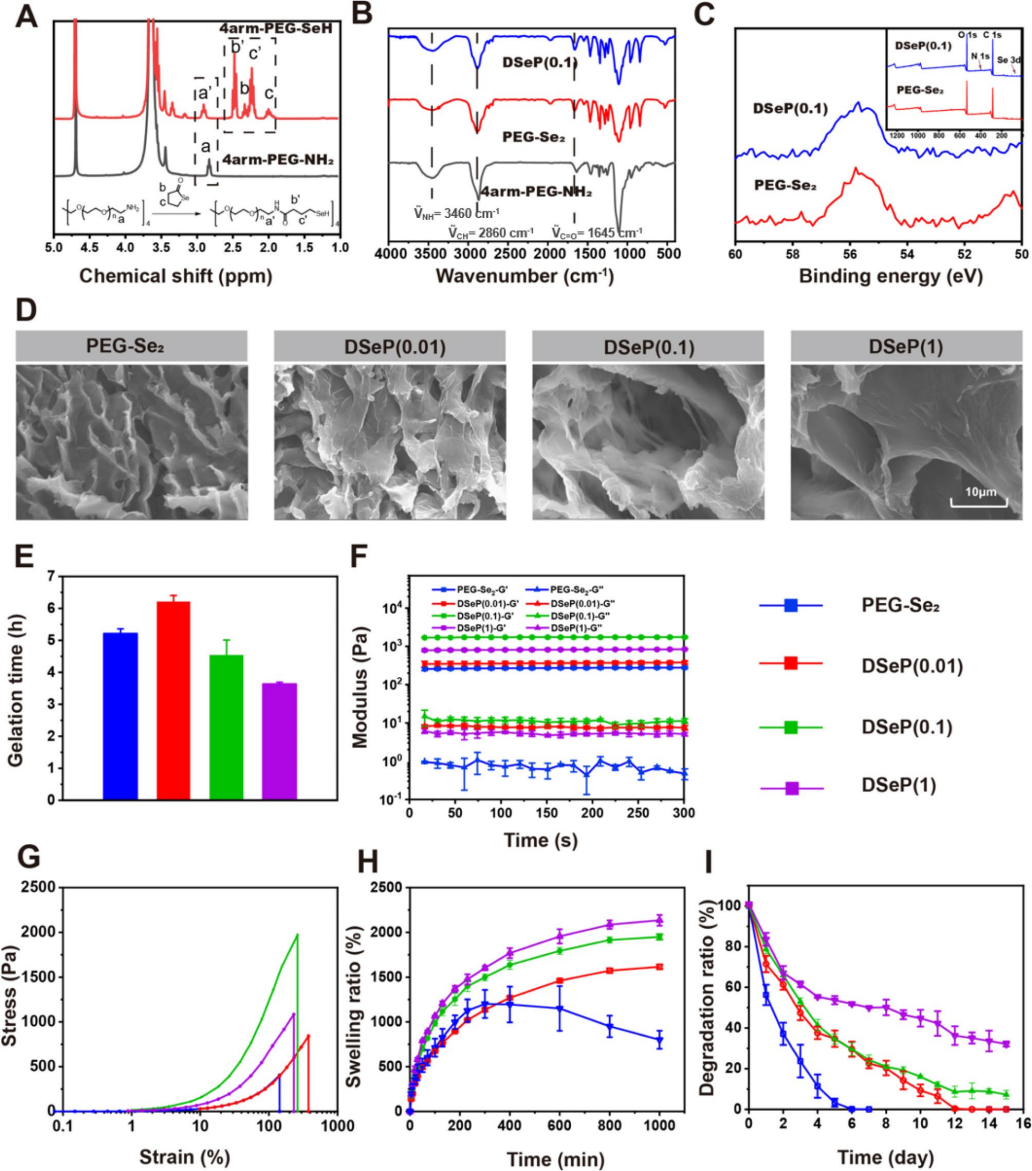
Characterization of the synthesized polymers and hydrogels. (A) ^1^ H NMR spectra of synthesized polymers. FTIR (B) and XPS spectra (C) of synthesized polymers and hydrogels. (D) SEM images of hydrogels. (E) Gelation times of hydrogels. (F) Rheological behaviors of hydrogels. (G) Stress–strain profiles of hydrogels by compression. (H) Swelling kinetics of hydrogels in PBS at pH 7.4 and 37 °C. (I) Degradation profiles of the hydrogels in PBS at pH 7.4 and 37 °C. Data are presented as the means ± SDs (n = 3)

PEG was selected as the key component of hydrogels owing to its high hydrophilicity, low immunogenicity, tunable physicochemical properties and ease of use [39]. 4-arm-PEG-NH_2_ is a multiarm PEG derivative that has amine groups attached to the terminals of each arm and is connected to one pentaerythritol core [25]. To endow 4-arm-PEG-NH_2_ with on-demand degradable properties, the γ-selenobutylacetone (γ-SBL) ring was opened and linked to 4-arm-PEG-NH_2_*via* a nucleophilic addition reaction to synthesize 4-arm-PEG-SeH (Fig. S1A) [24]. ^1^ H NMR analysis confirmed the successful synthesis of 4-arm-PEG-SeH (Fig. 1A). Signals that correspond to the protons of the -CH_2_-labeled terminal amino group shifted from approximately 2.82 ppm to 2.91 ppm, whereas peaks that were consistent with the protons of the ring-opened SBL presented at approximately 1.95 and 2.35 ppm, indicating the successful selenol modification of 4-arm-PEG-NH_2_. The calculated conversion rate of the protons of the -CH_2_-labeled terminal amino group was approximately 89%. Therefore, we successfully synthesized 4-arm-PEG-SeH with a one-pot procedure without using any additives or tedious steps. PEG-Se_2_ was then produced by oxidizing 4-arm-PEG-SeH with air (O_2_) in a stoichiometric ratio of 1:1 at ambient temperature, and the gelation of hydrogels was demonstrated by the inverted tube approach (Fig S1C and D).

We previously reported that diselenide bonds are too sensitive to maintain the rigid structure of hydrogels, and the mechanical strength of diselenide-crosslinked hydrogels can sharply decline in an aquatic environment [13]. Furthermore, the low adhesion property of PEG hydrogels is not conducive to wound healing. Hybrid hydrogels that integrate nanoparticles into their hydrogel networks through chemical interactions can be tailored to promote the mechanical and other desirable properties of PEG hydrogels [25]. PDANPs known to have enhanced hydrophilicity, anti-erosion capacity, and superior biochemical characteristics were incorporated into 4-arm-PEG-SeH to develop DSeP by means of Michael addition and crosslinking of 4-arm-PEG-SeH with PDANPs (Fig S1B, E and F). Simultaneously, the remaining selenol can be gradually oxidized under the action of oxygen to form diselenide. DSeP (0.01), DSeP (0.1) and DSeP (1) were defined as DSeP containing 0.01, 0.1 and 1 wt% PDANPs, respectively. The FTIR spectrum of DSeP exhibited enhanced peaks at 3460, 2860 and 1645 cm^-1^ corresponding to NH_2_, C-H and C = O, respectively, compared with the FTIR spectrum of PEG-Se_2_ formed through the oxidation of 4-arm-PEG-SeH without PDANPs (Fig. 1B). Compared with the XPS spectra of PEG-Se_2_, DSeP showed enhanced typical peaks corresponding to O and N (Fig. 1C). It is noteworthy that the peak corresponding to Se was at approximately 56 eV, and a sharp peak was detected in PEG-Se_2_, indicating that there is only one type of Se in the polymer. After the Michael addition reaction of the selenol of 4-arm-PEG-SeH with PDANPs, a mellow peak was detected in DSeP, signifying the incorporation of Se in the hydrogels. Altogether, the results verified the presence of PDANPs in DSeP.

With the aid of scanning electron microscopy (SEM), the surface morphology of the hydrogels was analyzed. SEM showed homogeneous pores with round shapes in all the hydrogels (Fig. 1D). The pore size of the hydrogels increased with the addition of PDANPs. In addition, the strong structural stability of the hydrogels was indicated by the pore structure integrity as well as its interconnectedness with the rest of the structure. When calculating the amount of time required for gelation, the point at which the curves representing storage modulus (G’) and loss modulus (G”) generated from the rheological test intersected was considered the gelation point. It was discovered that the time required for gelation is proportional to the weight ratio of PDANPs present in this system (Fig. 1E). The gelation time was the longest (6.3 ± 0.3 h) when 0.01 wt% PDANPs were incorporated into the hydrogels and gradually decreased to 4.6 ± 0.7 and 3.7 ± 0.1 h after the weight ratio of PDANPs was elevated to 0.1 and 1 wt%, respectively (Fig S2). The mechanical strength of DSeP was improved in a PDANP dose-dependent manner, suggesting that increasing PDANP concentrations in the hydrogel network might enhance gelation efficiency.

We speculate that PDANPs at a low concentration (0.01%) may hinder the oxidative coupling of diselenide in the hydrogels, thus prolonging the gelation time. However, when the concentration of PDANPs increases in DSeP, the possibility of reactivity between selenol and PDANPs increases, resulting in a shorter gelation time. The G’ and G” curves of the hydrogels were recorded over time to analyze the effect of varying PDANP weight ratios on the rheological characteristics of the hydrogels. The G´ of DSeP (0.1) (1500 Pa) was higher than that of DSeP (0.01) (220 Pa), DSeP (1) (750 Pa) and PEG-Se_2_ (190 Pa), suggesting that introducing a sizable quantity of PDANPs into the system may enhance the crosslinking density, thus increasing the G’ of hydrogels (Fig. 1F). The G’ and G’’ curves of DSeP over frequency and strain were also recorded (Fig. S3). These results indicate that the hydrogels can remain stable under high frequency and high strain conditions.

To determine the mechanical properties of the hydrogels, rotational stress‒strain measurements were taken. As depicted in Fig. 1G, the incorporation of PDANPs into PEG-Se_2_ effectively enhanced the mechanical properties of the hydrogels, and DSeP (0.1) exhibited the highest stress at the same strain. In particular, the increase in stress ranged from 400 to 2000 Pa at a strain of approximately 250% along with an increase in the PDANP weight ratio from 0 to 0.01, 0.1 and 1 wt%. Applying a strain of 100% throughout 50 loading‒unloading rotating test cycles facilitated the assessment of the recovery and resilience of the DSeP (Fig. S4). The recovered hydrogels showed stress‒strain curves that were similar to those of the original hydrogels following 50 loading‒unloading cycles, indicating that the hydrogels exhibited high fatigue resistance. Consequently, hydrogels with excellent mechanical properties can withstand stress without breaking, thus protecting surrounding tissues from damage.

Hydrogels are made of a network of polymers that can absorb large amounts of water and have a three-dimensional structure [1]. Because hydrogels may reduce wound effluence and lower the risk of infection, they are useful for accelerating the healing process. The swelling behavior of the hydrogels is illustrated in Fig. 1H. PEG-Se_2_ continuously absorbs water and gradually disintegrates into small fragments, making the swelling process of hydrogels difficult to measure owing to the weak dynamic covalent bonds of Se–Se in the hydrogels [13]. After 16 h, all hydrogels absorbed water to the maximum capacity, which varied between 1,550 and 2,000% of their starting weight. The lowest water absorption of approximately 1500% was observed in DSeP (0.01), followed by DSeP (0.1) and DSeP (1) (1900% and 2000%, respectively), demonstrating that the swelling rate increased when swelling equilibrium was reached as the concentration of PDANPs increased. After the addition of PDANPs, the hydrogels reached swelling equilibrium as a result of the reaction between selenol and PDANPs to generate a nondynamic monoselenide bond [25]. Therefore, the hydrogels can absorb water as much as 15–20 times their weight, which might aid in the removal of fluid from the wound area, thus speeding up the healing process.

Hydrogel disintegration at a rate that is suitable for biological uses is crucial [40]. The degradation rates of hydrogels were discovered to be adjustable in this study by changing the concentration of PDANPs (Fig. 1I). After approximately 1 week, PEG-Se_2_ showed the fastest degradation rate of 100%. With increasing concentrations of PDANPs, the weight ratios of DSeP (0.01), DSeP (0.1) and DSeP (1) were 22.3%, 24.3% and 50.0%, respectively. These findings suggest that increasing the mass ratio of PDANPs in the hydrogel network enhances the cross-linking efficiency and stability. Overall, the hydrogel DSeP(0.1) with the best mechanical properties and comprehensive properties was selected for subsequent experiments.

**Fig. 2:**
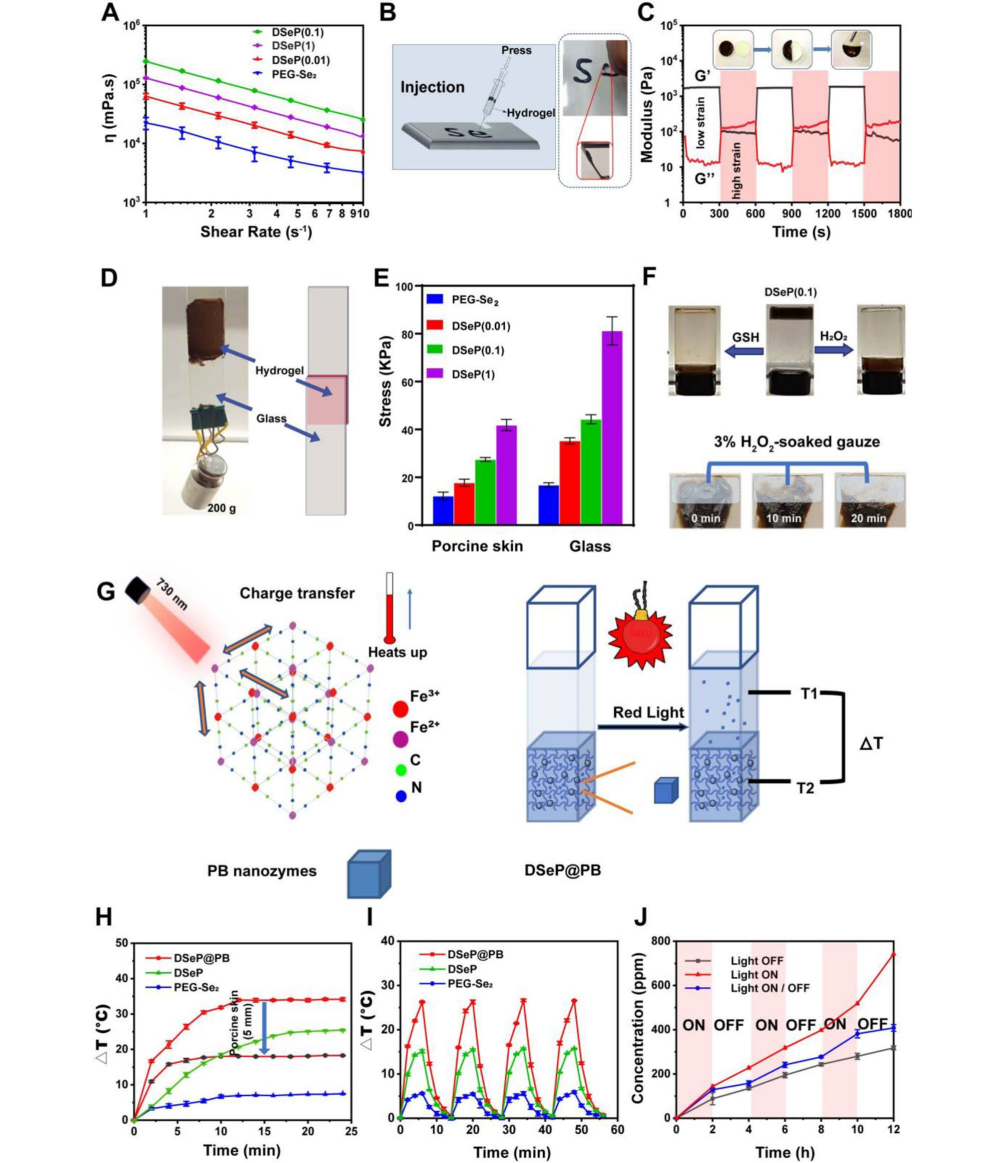
Mechanical properties and photobased properties of hydrogels. (A) The viscosity of hydrogels as a function of frequency. (B) Photographs of the injectability of DSeP(0.1). (C) The elastic modulus (G’) and viscous modulus (G’’) of DSeP(0.1) assessed by the strain sweep test from 1–1000% for 3 cycles. Photographs of the self-healing properties of the hydrogels: PEG-Se_2_ (transparent) and DSeP(0.1) (black). (D) Photographs of the adhesion of DSeP(0.1). (E) Adhesion strength of hydrogels. (F) Photographs of the on-demand degradability of DSeP(0.1) in the presence of 5% GSH and 3% H_2_O_2_. (G) Schema illustrating the photothermal mechanism of DSeP@PB. (H) Heating curves of the hydrogels. (I) Schema illustrating the heating and cooling curves of the hydrogels. (J) Release rate of PB nanozymes under different conditions (light on all times (red), light off all times (black), and light on and off alternatively (blue)). Data are presented as the means ± SDs (n = 3)

The injectability of hydrogels is critical for wound healing because injectable hydrogels can easily reach deep defects through a simple and minimally invasive procedure [41]. In this study, the hydrogels showed good stability over a range of frequencies from 1 Hz to 10 Hz and shear thinning properties (Fig. 2A), indicating the remarkable injectable property of the hydrogels. Injectability was also evaluated *via* simulation experiments, demonstrating that the hydrogels were readily injectable, could flow easily through a needle and could be recovered as stable hydrogels (Fig. 2B). Self-healing capability is another important property of hydrogels, and hydrogels with self-healing properties may repair themselves and return to their former form [42]. A rheology recovery test under various stresses was performed to examine the hydrogels’ self-healing abilities. The hydrogel network failed at a strain of approximately 800%, as determined by a strain amplitude sweep of DSeP. Subsequently, the rheological recovery tendency of the hydrogels was examined using a continuous step strain test (Fig. 2C). The recovered hydrogels experienced a dramatic drop in G’ from 1500 to 110 Pa after being subjected to a first high strain of 1000%. Moreover, G’’ was greater than G’, suggesting that the hydrogel network had collapsed. In the presence of a low strain (1%), the G’ of hydrogels rebounded to 1500 Pa, implying the partial restoration of the cross-linking. After another cycle of the test, the healed hydrogels displayed almost the same values of G’ and G’’ as the second cycle, demonstrating the self-healing of DSeP. The hydrogels also exhibited excellent self-healing ability after cutting and attaching. Altogether, these results suggest that, owing to chemical double cross-linking mechanisms (diselenide and selenide bonds), DSeP represents an ideal wound dressing capable of adapting to various irregularly shaped wounds.

An effective skin wound dressing requires both high adhesion performance and wound-repairing properties of the bioactive constituents of hydrogels [30]. The adhesive potential of the hydrogels was estimated using the tensile lap-shear test (Fig. 2D and E). The adhesive strength of the hydrogels markedly increased as the weight ratio of PDANPs increased. All hydrogels showed good adhesive strength between 20 and 80 kPa, which is considerably better than that of commercial dressings (approximately 5 kPa) [43]. These results indicate that DSeP has potential applications in wound protection. Possible mechanisms for hydrogel adhesion include catechol and quinone groups on DSeP reacting with amino or thiol groups on the protein through imide formation or Michael-type reactions [28].

Diselenide bonds are responsive to redox conditions [13]. Using this redox-dependent response, we observed the on-demand degradability of DSeP(0.1) under oxidative (H_2_O_2_) or reducing conditions (glutathione, GSH). As depicted in Fig. 2F, DSeP(0.1) could be dissolved in 15 min in the presence of H_2_O_2_ (3 wt%) or GSH (1 wt%). A gauze soaked in hydrogen peroxide (H_2_O_2_) was placed on half of the hydrogel covering a patch of porcine skin to simulate a dressing change. Complete dissolution of DSeP(0.1) occurred after 20 min of contact with the gauze. Similarly, the hydrogels were completely dissolved under reducing conditions (GSH), indicating that the hydrogels can be removed without causing any tissue damage. These results suggest that DSeP(0.1) has on-demand degradability and can be removed without damaging the tissue.

Selenium-containing hydrogels with on-demand degradation as attractive minimally invasive tools have a wide range of potential clinical applications [44]. However, typical selenium-containing hydrogels are usually mechanically weak. Unfortunately, the mechanical properties of hydrogels applied in wounds or tissue engineering are crucial because hydrogels inevitably tolerate mechanical forces through *in vitro* culture and *in vivo* healing [45]. In this work, reinforcement by PDANPs in the hydrogels provides mechanical strength and structural integrity, whereas the diselenide bonds help to maintain the dynamic characteristics. The idea of chemical double cross-linking mechanisms (diselenide and selenide bonds) may provide a general strategy to realize on-demand degradation and robust mechanical strength.

### Synthesis and characterization of DSeP@PB

PB nanozymes can degrade H_2_O_2_ and scavenge superoxides, thus exhibiting potent anti-inflammatory activity [34, 35, 46]. Under red light irradiation, PB nanozymes exhibit excellent photothermal properties, leading to the effective and rapid killing of bacteria [47, 48]. In this study, PB nanozymes were synthesized as described previously [37]. PB nanozymes had an average particle size of approximately 70 nm, with a small polydispersity index (PDI) of 0.2 and a zeta potential of -15.6 mV (Fig S5A). Transmission electron microscopy (TEM) was used to examine PB nanozymes and determine their morphology, revealing that the nanozymes had a cubical shape with a diameter of 30 nm (Fig S5B). The discrepancy between DLS and TEM measurements is attributed to the following reason: DLS represents the hydrodynamic size, whereas TEM indicates the dried size of nanoparticles. The FTIR (Fig S5C) and UV‒vis absorbance (Fig S5D) spectra revealed the typical characteristic peaks of PB nanozymes, indicating the successful synthesis of PB nanozymes.

Furthermore, PB nanozymes were embedded in DSeP to fabricate hybrid hydrogels termed DSeP@PB. Compared with DSeP, the FTIR spectra of DSeP@PB (Fig S6A) showed the typical peak corresponding to the CN group at 2090 cm^-1^. Compared with DSeP, the XPS spectra of DSeP@PB (Fig S6B) showed that the peak corresponding to Se was wider, indicating the interaction of selenium with PB nanozymes. Energy dispersion spectroscopy (EDS) was used to characterize the elements on the surface of the hydrogels (Fig S6C–D). As depicted in Fig S6E–F, iron and selenium were distributed in a uniform manner on the surface of the hydrogels, suggesting that PB nanozymes were uniformly incorporated into the hydrogels. Altogether, these results verified the presence of PB nanozymes in DSeP@PB.

### Photothermal properties and controllable release of hydrogels

Owing to the presence of PB nanozymes in DSeP@PB, we speculated that the hydrogels can exhibit photothermal properties under 730-nm red light irradiation (Fig. 2G) [48]. As expected, the temperature of PEG-Se_2_ only showed a moderate increase under 730-nm red light irradiation (Fig. 2H), whereas that of DSeP eventually increased by 25 °C after 20 min. Notably, after the incorporation of PB nanozymes, DSeP@PB exhibited better photothermal properties, and their temperature significantly increased by 35 °C after irradiation for 12 min. These findings illustrated that the introduction of PDANPs and PB nanozymes synergistically enhanced the photothermal activity of PEG-Se_2_. To simulate *in vivo* conditions, the photothermic property of DSeP@PB was evaluated on 5-mm-thick porcine skin. The temperature of the hydrogels increased by 17 °C and achieved equilibrium. At this temperature, the antibacterial activity could be maintained without harming normal body tissues [48]. Furthermore, the photothermal stability of the hydrogels was evaluated through light switch experiments and several photothermal cycles (Fig. 2I). After four ON/OFF laser cycles, there was almost no change in photothermal performance, indicating the high photothermal stability of DSeP@PB.

Numerous stimuli-responsive hydrogels designed for controlled drug release are excellent platforms because they can recognize stimuli such as light or temperature and can be triggered to release drugs [16, 49]. Considering that light’s wavelength, intensity, exposure area, and time could be remotely tuned for “on-demand” regulated drug delivery, using light as an external stimulus could provide precise spatiotemporal regulation of drug release [13]. We determined whether light could trigger the release of PB nanozymes from DSeP@PB. As illustrated in Fig. 2J, in the absence of light irradiation, PB nanozymes were released slowly from DSeP@PB, whereas constant light irradiation triggered a faster release of PB nanozymes. Importantly, the release of PB nanozymes from DSeP@PB under 2-h light/dark cycles showed a light-triggered pattern. The release rate of PB nanozymes was significantly higher in the 2-h light irradiation cycle than in the 2-h dark cycle. We speculated that the light-controllable release of hydrogels was attributed to the light-triggered dynamic exchange of diselenide bonds and photothermal effect-induced enhanced drug release [23, 50]. Altogether, these findings highlighted the light-triggered sustained release of PB nanozymes from DSeP.

### Antibacterial activity of DSeP@PB *in vivo* and *in vitro*

**Fig. 3:**
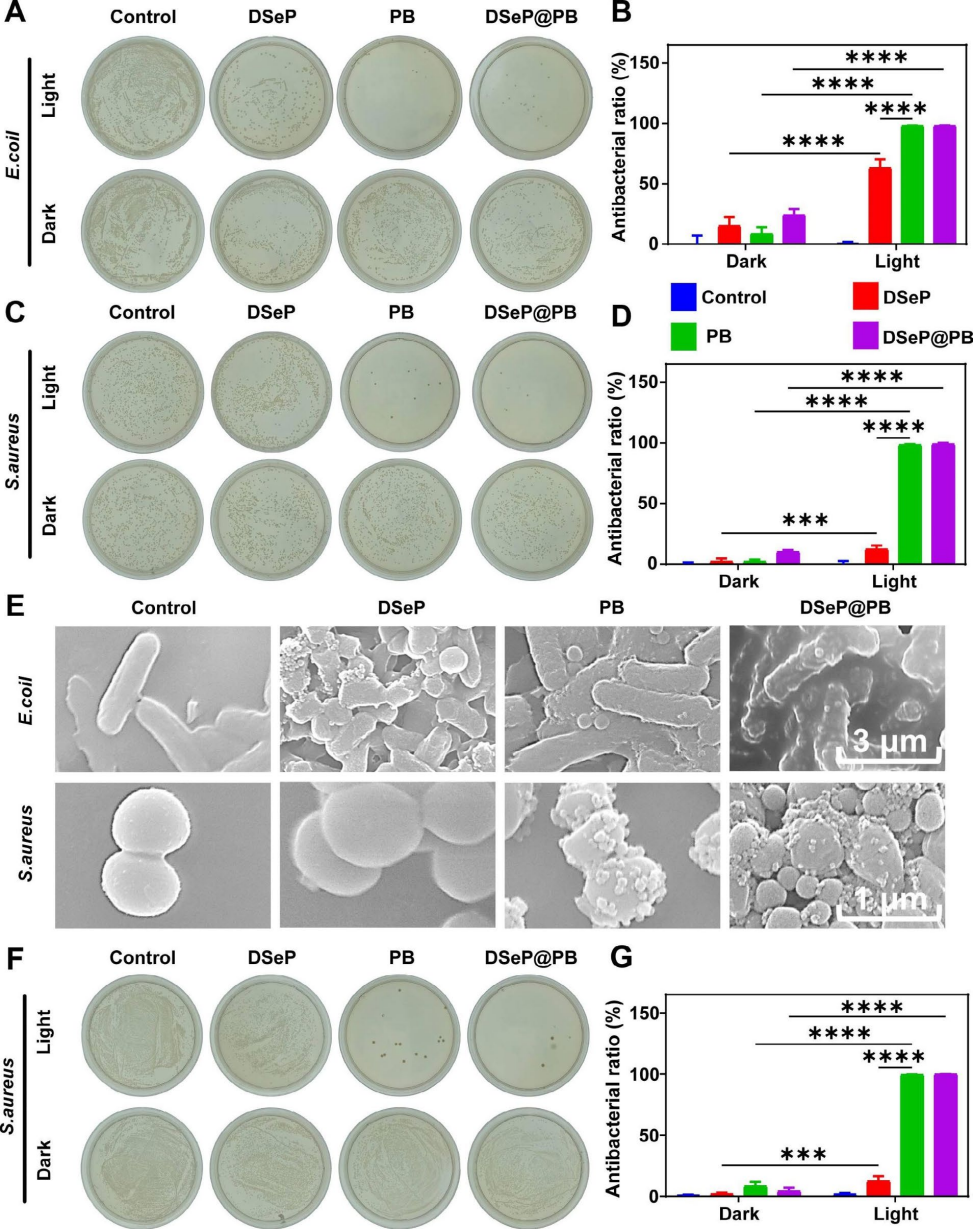
Antibacterial activity of hydrogels and PB nanozymes. (A) and (C) Photographs of plates showing the bacterial colony formation assay in the presence or absence of red light irradiation. (B) and (D) Antibacterial ratio of bacterial colonies calculated from (A) and (C), respectively. (E) Representative field emission scanning electron microscopy (FE-SEM) images of bacteria after treatment. (F) Photographs of plates showing the bacterial colony formation assay after the *S. aureus* from the mice was inoculated in the culture plates. (G) Antibacterial ratio of bacterial colonies calculated from (F). *P < 0.05; **P < 0.01; ***P < 0.001; ****P < 0.0001; Data are presented as the means ± SDs (n = 3)

Colony formation assays in spread plates were conducted to assess the capacities of hydrogels and PB nanozymes as antibacterial agents against *S. aureus* and *E. coli* (Fig. 3A–D). Under conditions devoid of red light, the antibacterial efficiency of DSeP, PB nanozymes and DSeP@PB against *E. coli* was 15% ± 7%, 9% ± 5% and 24% ± 5%, respectively, and that against *S. aureus* was 2% ± 3%, 2% ± 2% and 10% ± 1%, respectively, indicating their weak antibacterial ability against *S. aureus* and *E. coli*. Following 15 min of 730-nm red light irradiation, the antibacterial efficiency of DSeP, PB nanozymes and DSeP@PB against *E. coli* was significantly increased, reaching up to 63% ± 7%, 98% ± 1% and 98% ± 1%, respectively. In addition, the antibacterial efficiency of DSeP, PB nanozymes and DSeP@PB against *S. aureus* was 12% ± 3%, 98% ± 1% and 99% ± 1%, respectively. These findings illustrate that both DSeP@PB and PB nanozymes can effectively fight against both *S. aureus* and *E. coli* when subjected to red light irradiation.

SEM was used to evaluate changes in bacterial structure and integrity following treatment with hydrogels (Fig. 3E). After being exposed to red light for 15 min, both *S. aureus* and *E. coli* retained their normal shapes (the control group). After treatment with DSeP, the morphology of *S. aureus* and *E. coli* was also hardly altered, with only minor structural damage in the body’s periphery. Following treatment with PB nanozymes and DSeP@PB, both *E. coli* and *S. aureus* showed serious deformations and shrinking, indicating that the bacteria were damaged after light irradiation. Based on the promising antibacterial efficacy of PB nanozymes and DSeP@PB *in vitro*, their *in vivo* therapeutic efficacy was evaluated in wound infections caused by *S. aureus*. A majority of wound infections are caused by the gram-positive bacterium *S. aureus*; therefore, a mouse model of wound infection caused by *S. aureus* was established [51, 52]. Each mouse had a wound 15 mm in diameter carved off its back. Wounds then received various posttreatment care after bacterial solution was added to the wounds. Bacterial counts in *S. aureus*-infected wounds were evaluated using the colony formation-based spread plate method. As depicted in Fig. 3F and G, no significant differences were observed in *S. aureus* counts (*P* > 0.05) among all the groups without light irradiation. After light irradiation, the DSeP@PB and PB groups showed excellent antibacterial efficiency (DSeP@PB: 99% ± 1%, *P* < 0.0001; PB nanozymes: 99% ± 1%, *P* < 0.0001), which is consistent with their superior antibacterial activity observed *in vitro*. Altogether, these results demonstrate that both DSeP@PB and PB nanozymes exhibit superior antibacterial performance under 730-nm light irradiation.

### Biocompatibility and antioxidant efficacy of Hydrogels and PB nanozymes

*Via* distinct redox pathways, PB nanozymes replicate the actions of catalase, peroxidase, and superoxide dismutase (SOD) [36, 38]. Consequently, the xylenol orange test was used to analyze the PB nanozyme capacity for the total degradation of H_2_O_2_. The results illustrated that PB nanozymes could degrade H_2_O_2_ in a time- and concentration-dependent manner (Fig S7A). Although a low concentration of PB nanozymes (25 µg·mL^-1^) had minimal effects on the degradation rate of H_2_O_2_, PB nanozymes at higher concentrations led to a significant enhancement of the degradation rate. In the presence of 50 µg·mL^-1^ PB nanozymes, approximately 23% ± 9% of H_2_O_2_ was degraded within 60 min, and by the 120-min mark, the rate of degradation had reached 53% ± 3%. In the presence of 100 µg·mL^-1^ PB nanozymes, the degradation rate of H_2_O_2_ was closer to 100% after 60 min. Furthermore, potent superoxide-scavenging capacity was shown in PB nanozymes in a dose- and time-dependent manner (Fig S7B). PB nanozymes (10 µg·mL^-1^) scavenged 3% ± 1% and 5% ± 2% superoxide radicals within 30 and 60 min, respectively. At a dosage of 100 µg·mL^-1^, PB nanozymes showed the strongest superoxide-scavenging capability, with removal rates of 26% ± 4%, 33% ± 5% and 43% ± 3% at 30 min, 60 and 120 min, respectively. Altogether, these findings proved that PB nanozymes are effective ROS scavengers.

**Fig. 4:**
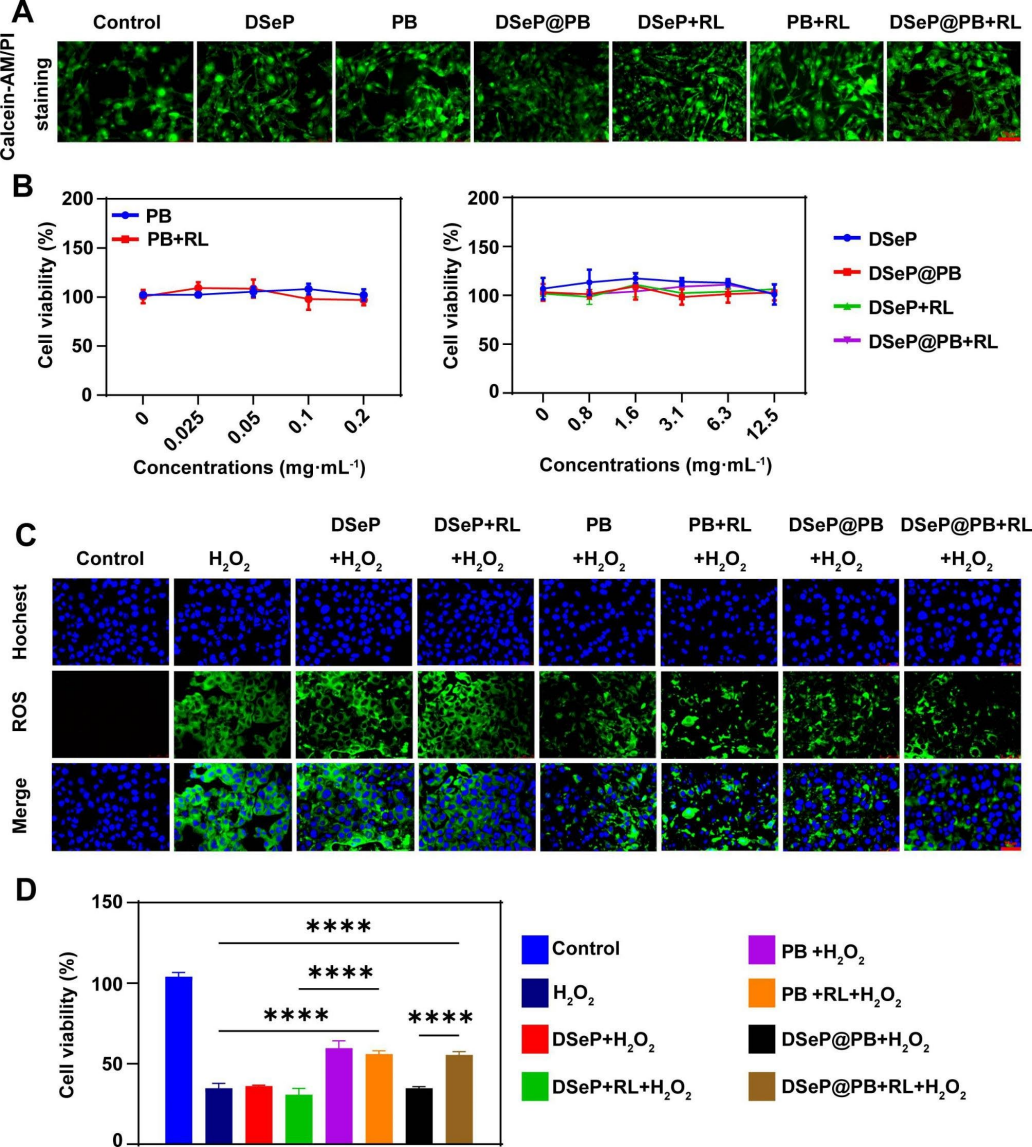
*In vitro* biocompatibility and antioxidant effect of hydrogels and PB nanozymes. (A) NIH-3T3 cells were stained with calcein-AM (green) and propidium iodide (PI, red) after different treatments for 24 h. Bars represent 75 μm. (B) Cell viability of NIH-3T3 cells evaluated by the MTT assay after different treatments for 24 h. (C) ROS fluorescence images of NIH-3T3 cells after different treatments. Bars represent 75 μm. D) Cell viability of NIH-3T3 cells evaluated by the MTT assay after different treatments. *P < 0.05; **P < 0.01; ***P < 0.001; ****P < 0.0001. RL (red light) indicates 730 nm red light irradiation. Data are presented as the means ± SDs (n = 3)

Furthermore, the biocompatibility of DSeP@PB and PB nanozymes was evaluated using NIH-3T3 fibroblasts. As shown in Fig. 4A, live/dead staining with calcein-AM/PI did not show a significant loss of cell viability or changes in cell morphology in cells incubated with PB nanozyme and DSeP@PB (Fig. 4B). These results demonstrated that the hydrogels and PB nanozymes had a negligible impact on cell viability, which is in line with the results of the research by Han et al. [37]. We speculate that when suspended in growth media, the hydrogels and PB nanozymes did not damage the cells. Even after being subjected to 730-nm red light, the cells may have been damaged but could quickly recover to normal growth over time.

The capacity of PB nanozymes and hydrogels to scavenge ROS was verified by measuring the levels of intracellular ROS using a fluorescent probe named dihydrofluorescein diacetate (DCFH-DA) (Fig. 4C). As anticipated, treatment with H_2_O_2_ resulted in a considerable increase in the intracellular fluorescence signal, which indicated a remarkable increase in ROS generation in the treated cells. The level of intracellular ROS was reduced in the DSeP-treated group, indicating that DSeP had certain antioxidant properties. Notably, in the presence of PB nanozymes and DSeP@PB, the fluorescence signal dramatically decreased, suggesting that PB nanozymes and DSeP@PB had efficient ROS-scavenging activity. The *in vitro* cytoprotective effects of PB nanozymes and DSeP@PB were examined in the presence of H_2_O_2_ (Fig. 4D). As expected, cell viability significantly decreased to 35% ± 3% after H_2_O_2_ treatment. However, DSeP did not affect cell viability, implying minimal cytoprotective effects. Furthermore, after the addition of PB nanozymes, cell viability markedly increased to 60% ± 5%. Although 730-nm light irradiation did not affect the cytoprotective effects of PB nanozymes and DSeP, it significantly promoted the cytoprotective effects of DSeP@PB (*P* < 0.05), suggesting that light-triggered release of PB nanozymes contributes to the cytoprotective effects of DSeP@PB + RL (DSeP@PB combined with red light irradiation). This confirmed our hypothesis that DSeP@PB + RL showed improved resistance to oxidative stress by scavenging exogenous oxidants.

### *In vivo* wound healing in a diabetic mouse model

To assess the *in vivo* toxicity of the hydrogels and PB nanozymes, the body weight, blood biochemical parameters and histological characteristics were investigated in healthy ICR mice. The major organs of all treated mice exhibited no pathological changes, demonstrating that the hydrogels and PB nanozymes had no systemic toxicity (Fig S8A). In addition, the biochemical analysis showed that each index was within the normal range, indicating that the hydrogels and PB nanozymes had no systemic toxicity *in vivo* (Fig S8B). Administration of the hydrogels and PB nanozymes had a negligible influence on the body weight of mice, which increased steadily during the 21-day follow-up (Fig S8C). These results indicate that the hydrogels and PB nanozymes serve as an effective and safe wound dressing to promote wound healing.

**Fig. 5:**
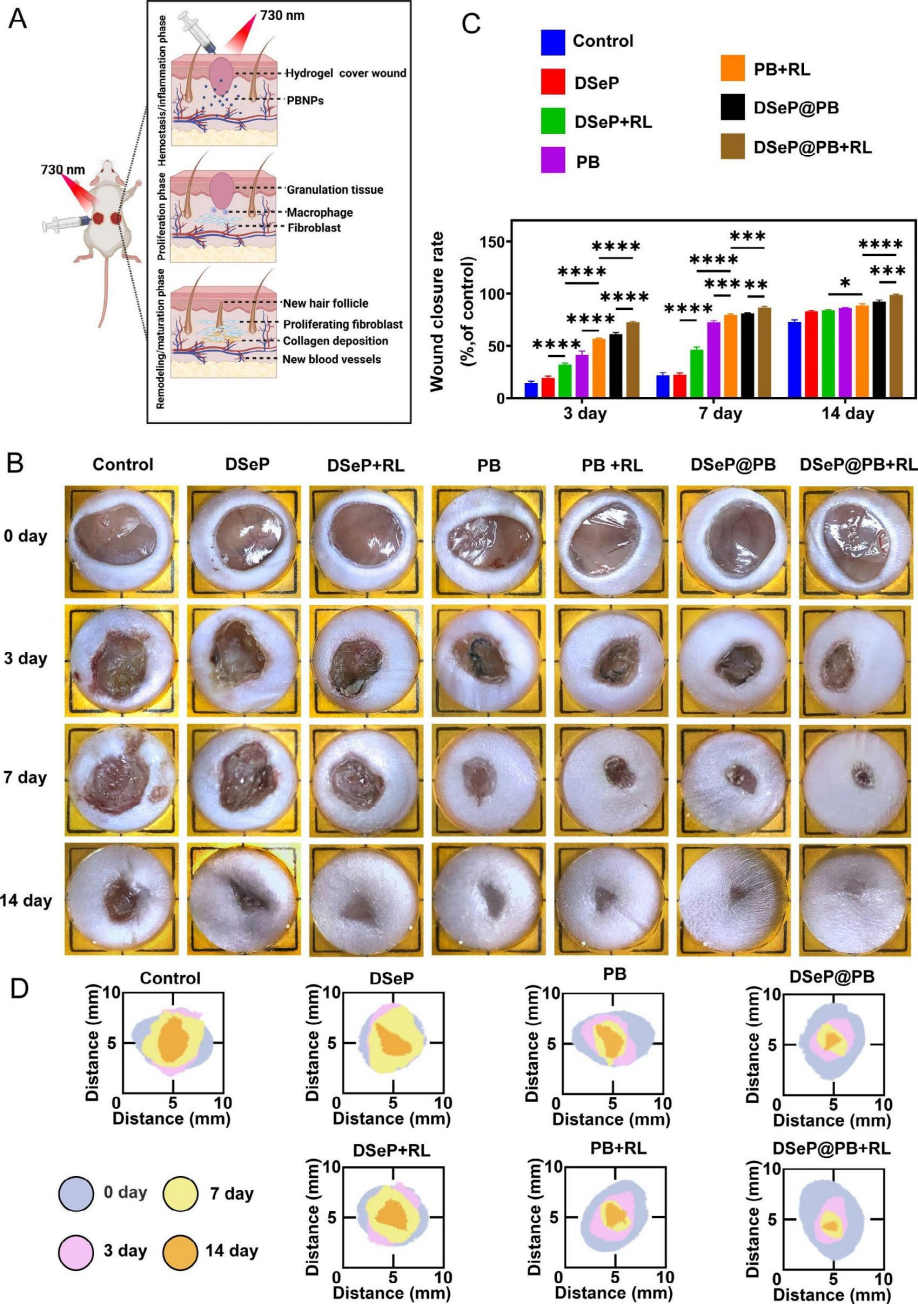
Wound healing of diabetic wounds promoted by hydrogels and PB nanozymes. (A) Schematic diagram of the wound-healing mechanism in diabetic wounds in mice. (B) Representative images of the wound healing process of mice with each treatment at different time points. (C) Wound closure rates of mice with each treatment at different time points. (D) Simulation diagram of the wound healing process. *P < 0.05; **P < 0.01; ***P < 0.001; ****P < 0.0001. Data are presented as the means ± SDs (n = 3)

The *in vitro* studies demonstrated that DSeP@PB is a promising candidate as a wound dressing for chronic diabetic wounds. Therefore, a diabetic mouse model was used to assess the healing-acceleration performance of the hydrogels (Fig. 5A). The wound area in all seven groups decreased with an increase in postoperative time (Fig. 5B–D). DSeP, PB nanozymes and DSeP@PB exhibited accelerated wound closure rates in the presence of 730-nm light irradiation compared with the absence of irradiation. Compared with DSeP and PB nanozymes, DSeP@PB exhibited significant improvement in wound closure, indicating that DSeP and PB nanozymes have synergistic effects on wound healing. In the control group, wounds were still open on day 14 but were completely closed in the DSeP@PB + RL group. The quantitative examination of the wound site illustrated that wounds treated with DSeP@PB + RL had healing ratios of 99% ± 1%, which differed substantially from that of the control group (73% ± 1%) (*P* < 0.05), indicating that DSeP@PB and red light irradiation synergistically accelerated wound healing. Altogether, the results demonstrate that DSeP@PB + RL has superior wound healing ability compared to the other groups.

### Histomorphological evaluation

**Fig. 6:**
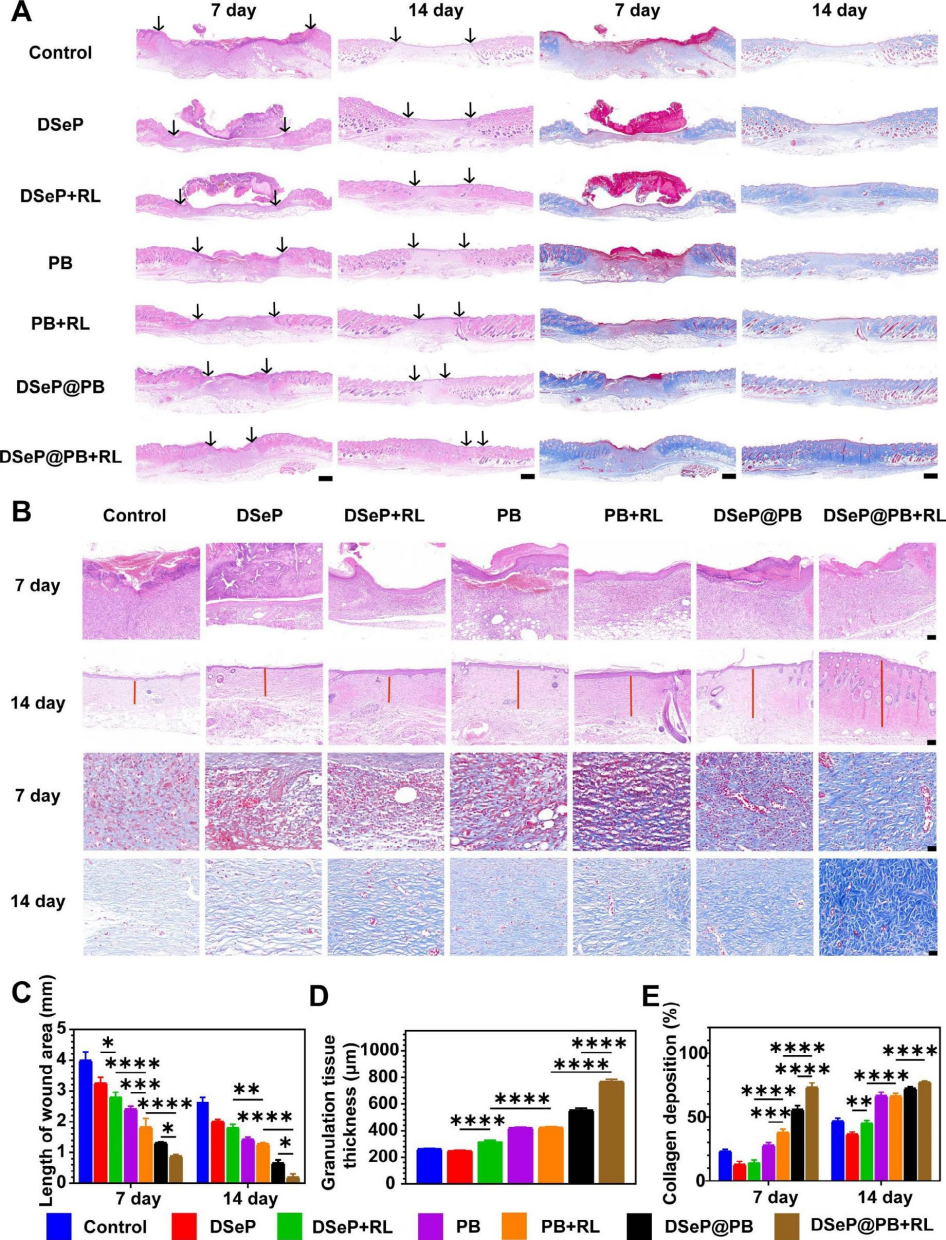
Histological analysis of diabetic wounds treated with hydrogels and PB nanozymes. (A) Representative H&E and Masson staining images of diabetic wounds of mice with each treatment at days 7 and 14. Scale bars represent 500 μm. (B) H&E and Masson staining images of diabetic wounds of mice with each treatment at days 7 and 14. Bars represent 100 μm. (C) Quantification of the length of the wound area at days 7 and 14. (D) Quantification of granulating tissues at day 14. (E) Quantitative analysis of collagen deposition at days 7 and 14. *P < 0.05; **P < 0.01; ***P < 0.001; ****P < 0.0001. Data are presented as the means ± SDs (n = 3)

In diabetic wounds, histological examination was carried out to analyze the level of regenerated skin quality (Fig. 6A and B). The wound site was stained with H&E and Masson for the purpose of determining the extent of granulation tissue, the thickness of the epidermis, and the number of blood vessels, along with characterizing the regenerated skin layers. As depicted in Fig. 6C, wound length was assessed in the treatment groups on days 7 and 14. All treatment groups had shorter wounds than the control group (2.7 mm ± 0.1 mm), with the DSeP@PB + RL (0.2 mm ± 0.1 mm) group having the shortest wounds (*P* < 0.05).

In injuries accompanied by inflammation, parenchymal cells cannot complete the healing process on their own [53]. For wounds of this kind, granulation tissue is a crucial component of the healing process. It proliferates at first, then degrades and absorbs foreign bodies and necrotic tissues to fill up the wound, and finally transforms into scar tissue to seal it [6]. Therefore, the development of denser granulation tissue throughout the healing process is a crucial sign for gauging the success of wound repair. As depicted in Fig. 6B and D, all treated groups had denser granulation tissue than the control group on day 14 (*P* < 0.05), with the tissue being thickest in the DSeP@PB + RL group. These results indicated that DSeP@PB + RL irradiation had the best wound-healing effects.

Successful wound healing relies on the deposition of collagen. As the fibrin-fibronectin clot that forms at the initial stage of wound healing is gradually replaced by collagen, the wound becomes stronger and more resistant to further damage [7]. The cells that contribute to angiogenesis and connective tissue development use collagen as a scaffold upon which to adhere, proliferate, and differentiate [54]. Figure 6B and E show the deposition of newly produced collagen in wounds. On day 14, the control group demonstrated sparse and disorganized low-level collagen deposition; however, the treated groups, especially the DSeP@PB + RL group, exhibited considerably increased deposition of collagens compared with the control group. Wounds subjected to DSeP@PB + RL showed satisfactory healing, as determined by H&E and Masson staining, with a quick healing rate and collagen synthesis at the wound site.

**Fig. 7:**
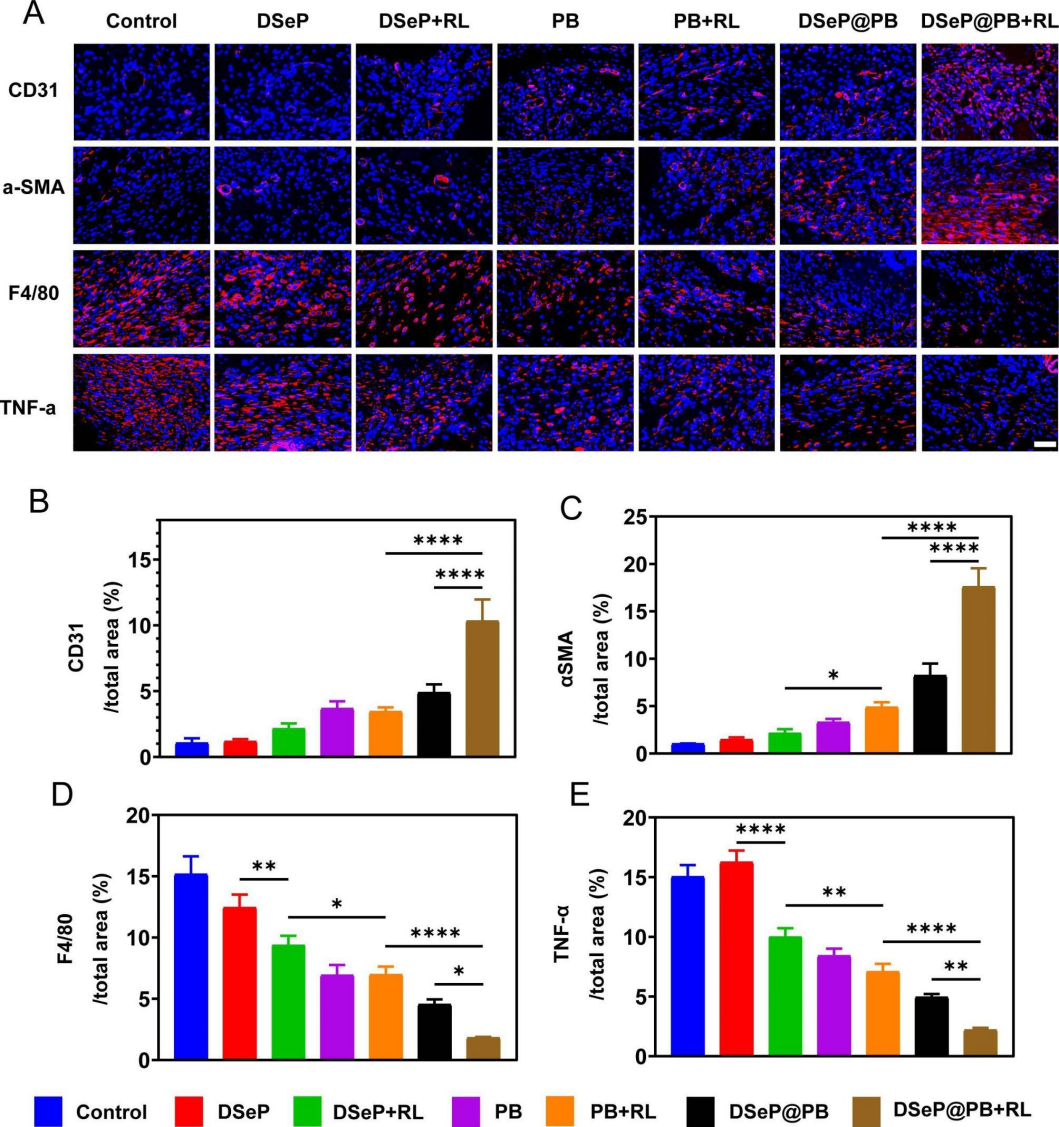
Immunofluorescence analyses of diabetic wounds treated with hydrogels and PB nanozymes. (A) Photographs of CD31 (red) with DAPI (blue), α-SMA (red) with DAPI (blue), F4/80 (red) with DAPI (blue) and TNF-α (red) with DAPI (blue) in sections of diabetic wounds. (B) - (E) Quantification of immunohistochemistry analysis. *P < 0.05; **P < 0.01; ***P < 0.001; ****P < 0.0001. Data are presented as the means ± SDs (n = 3)

Furthermore, the effects of hydrogels and PB nanozymes on angiogenesis were investigated *in vivo* (Fig. 7A). To detect the presence of new blood vessels, the vessel walls were stained with immunofluorescence antibodies against CD31 (a marker unique to the vascular endothelium) and α-SMA (a marker specific to vascular smooth muscle cells). As illustrated in Fig. 7B and C, the expression of CD31 and α-SMA was highest in the DSeP@PB + RL group, followed by the DSeP@PB group. These findings suggest that DSeP@PB and red-light irradiation may function together to generate robust angiogenesis and neovascularization, thus promoting skin re-epithelialization, collagen deposition, and cutaneous maturation, all of which are necessary for wound closure.

We speculated that topical administration of DSeP@PB may reduce the recruitment of macrophages at the wound site since these hydrogels can serve as both ROS scavengers and anti-inflammatory mediators *in vitro*. Immunostaining for F4/80 glycoprotein, which is produced by macrophages and is often used to detect tissue macrophages, facilitated us in evaluating the magnitude of macrophage recruitment [35]. As depicted in Fig. 7D, DSeP showed a minimal effect on the proportion of F4/80-positive cells, whereas DSeP + RL substantially reduced the proportion of F4/80-positive cells, suggesting that 730-nm light irradiation could reduce macrophage infiltration in the DSeP group. The decrease observed in macrophage infiltration in the PB, PB + RL and DSeP@PB groups may result from the potential anti-inflammatory action of nanozymes. Importantly, the proportion of infiltrating macrophages in the DSeP@PB + RL group was the lowest, being almost 8 times lower than that in the control group. Immunostaining was performed to evaluate the expression of TNF-α, which is implicated in the first stages of inflammatory processes and is released by activated macrophages as a proinflammatory cytokine [55, 56] As displayed in Fig. 7E, the DSeP + RL group had considerably lower TNF-α expression levels than the DSeP group, suggesting that 730 -nm light irradiation could reduce the expression of TNF-α in the DSeP group. Similarly, PB, PB + RL, and DSeP@PB all showed reduced TNF-α expression, which may be attributable to the potential anti-inflammatory effect of nanozymes. The expression of TNF-α in the DSeP@PB + RL group was the lowest, being almost 7 times lower than that in the control group. Therefore, DSeP@PB + RL showed the best *in vivo* anti-inflammatory effects to reduce the macrophage burden and prevent the secretion of proinflammatory cytokines in diabetic wounds.

### Gene expression analysis

**Fig. 8:**
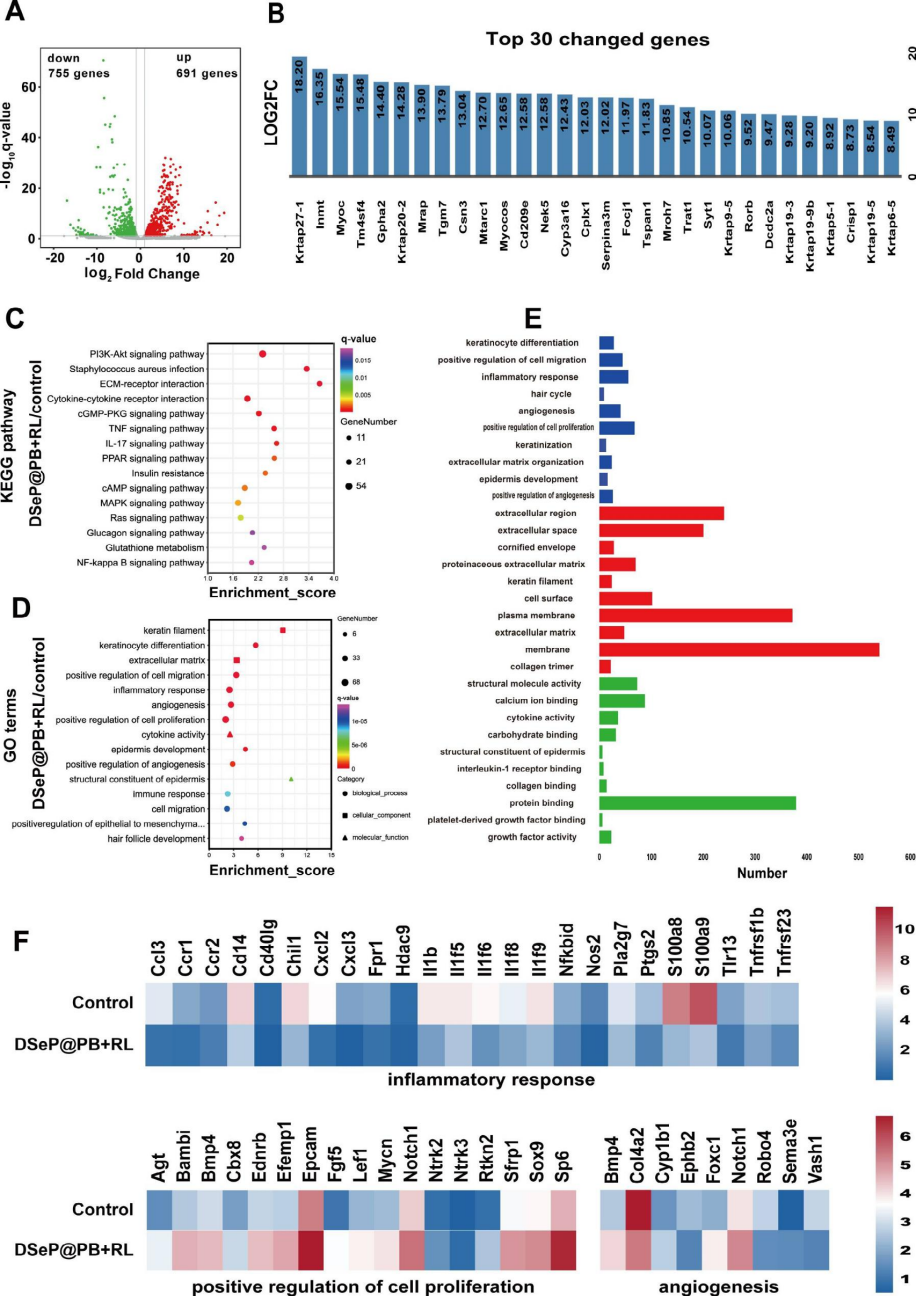
RNA-seq assessments of diabetic wounds at day 14 after treatment with DSeP@PB + RL. (A) Volcano plot of significant gene expression changes in the DSeP@PB + RL/control comparison. (B) Genes involved in immune regulation as measured by GO classification analysis and a list of the top 30 upregulated genes and corresponding log2Fc (log2-fold change). (C) KEGG pathway enrichment analysis of the DSeP@PB + RL/control comparison. (D) GO classification analysis of the DSeP@PB + RL/control comparison. (E) Gene expression involved in BP, CC and MF was quantified. (F) Gene expression analysis of the inflammatory response, angiogenesis and positive regulation of cell proliferation in diabetic wounds, with data normalized to the control group (without treatment). n = 3 mice per treatment

RNA-seq was performed to analyze gene expression in tissues surrounding diabetic wounds, using the untreated group as the control. Using the empirical Bayes method (fold change ≥ 4; *q*-value < 0.05), 1446 significant differentially expressed genes (DEGs) were discovered in the DSeP@PB + RL group, with 691 upregulated and 755 downregulated genes. The distribution of these DEGs was visualized on volcano plots (Fig. 8A). A list of the 30 most upregulated genes is presented in Fig. 8B.

When comparing the DSeP@PB + RL and control groups, KEGG pathway enrichment analysis revealed that a substantial number of DEGs in the DSeP@PB + RL group were enriched in pathways related to inflammatory reactions, including the NF-kappa B, IL-17, and TNF signaling pathways, as well as cytokine‒cytokine receptor interactions (Fig. 8C). Subsequently, Gene Ontology (GO) analysis was performed on DEGs, and the results were significantly different between the DSeP@PB + RL and control groups (Fig. 8D). More genes in the DSeP@PB + RL group than in the control group were found to be associated with molecular functions (MFs), cell components (CCs), and biological processes (BPs), such as inflammatory responses, angiogenesis, positive modulation of cell proliferation, epidermis development, cell migration, and growth factor activity, which are related to skin regeneration (Fig. 8E). The expression of inflammatory factors, including *Il-1b*, *Ccl3* and *Nos2*, which are detrimental for skin regeneration, was decreased in the DSeP@PB + RL group compared with the control group. The findings herein are in line with those of *in vivo* immunofluorescence studies (Fig. 8F). Genes involved in the positive control of cell proliferation, such as *Agt, Bambi, Bmp4, Ednrb, Efemp1*, and *Fgf5*, were more highly expressed in the DSeP@PB + RL group. In addition, genes involved in the positive modulation of angiogenesis, such as *Cyp1b1*, *Foxc1*, *Notch1*, and *Sema3e*, were expressed at higher levels in the DSeP@PB + RL group, whereas genes involved in the negative modulation of angiogenesis, such as *Col4a2*, *Ephb2*, *Robo4*, and *Vash1*, were expressed at lower levels.

Taken together, these findings illustrated that DSeP@PB combined with red light irradiation offers a promising strategy for diabetic wound healing. Our data help to elucidate the mechanism underlying the wound healing-promoting efficacy of DSeP@PB. After DSeP@PB is injected into diabetic wounds, PB nanozymes are triggered by RL to be released from the hydrogels under red light irradiation, exerting potent anti-inflammatory and antioxidant effects by scavenging exogenous oxidants. Finally, DSeP@PB + RL showed the most efficient wound healing activity by inhibiting inflammation and stimulating angiogenesis and collagen deposition.

## Conclusion

A promising selenide-linked polydopamine-reinforced hybrid hydrogel, DSeP@PB, with controllable degradation and nanozyme release, was developed as a new dressing material to promote diabetic wound healing. The hydrogels were fabricated *via* reinforcement of selenol-end capping PEG hydrogels by PDANPs and PB nanozymes in a one-pot approach by chemical double cross-linking mechanisms (diselenide and selenide bonds), which resulted in the hydrogel displaying on-demand degradation, excellent injectability, and ideal mechanical strength. Notably, DSeP@PB showed the most efficient wound healing activity by stimulating angiogenesis and collagen deposition and inhibiting inflammation. In addition to displaying immunoregulatory functions and mechanical strength, the hydrogel was also shown to be biocompatible and easily synthesized. Taken together, the hybrid hydrogel displays promise as a new dressing material for the management of diabetic wounds.

## Electronic supplementary material

Below is the link to the electronic supplementary material.

Supplementary Material 1

## Data Availability

The datasets used and/or analyzed during the current study are available from the corresponding author on reasonable request.

## Abbreviations

PEG: Polyethylene glycol
PDANPs: Polydopamine nanoparticles
PB: Prussian blue
ROS: Reactive oxygen species
DTT: Dithiothreitol
PEtOx: Poly(2-ethyl-2-oxazoline)
4-arm-PEG-SeH: 4-arm polyethylene glycol (PEG) with selenol
PEG-Se_2_: Diselenide-containing polyethylene glycol hydrogels
DSeP: PEG-Se_2_ loaded with PDANPs
DSeP@PB: PEG-Se_2_ loaded with PDANPs and PB nanozymes
FTIR: Fourier transform infrared spectrometer
XPS: X-ray photoelectron spectroscopy
SEM: Scanning electron microscopy
G': Storage modulus
G": Loss modulus
GSH: Glutathione
DLS: Dynamic light scattering
TEM: Transmission electron microscope
UV‒vis: Ultraviolet and visible spectrophotometry
EDS: Energy dispersion spectroscopy
SOD: Superoxide dismutase
PI: Propidium iodide
MTT: Methylthiazoletetrazolium
RL: Red light
H_2_O_2_: under oxidative
ROS: reactive oxidative species
DCFH-DA: Dichlorodihydrofluorescein diacetate
UA: Uric acid
AST: Aspartate aminotransferase
ALT: Alanine aminotransferase
PFA: Paraformaldehyde
H&E: Hematoxylin and eosin
STZ: Streptozotocin
FBS: Fetal bovine serum
PBS: Phosphate-bufered saline
GO: Gene ontology
KEGG: Kyoto Encyclopedia of Genes and Genomes
DEGs: differentially expressed genes
MFs: molecular functions
CCs: cell components
BPs: biological processes.
