# Supplementary material for: Selenide-linked polydopamine-reinforced hybrid hydrogels with on-demand degradation and light-triggered nanozyme release for diabetic wound healing
Source: Biomater Res. 2023 May 18;27:49. doi: 10.1186/s40824-023-00367-w (PMC10193707; doi:10.1186/s40824-023-00367-w)
Supplement: Supplementary file 1 — Supplementary Material 1 [file 40824_2023_367_MOESM1_ESM.doc]

**Supporting information**

**Selenide-linked Polydopamine-Reinforced Hybrid Hydrogels with On-demand Degradation and Light-triggered Nanozyme Release for Diabetic Wound Healing**

**EXPERIMENTAL SECTION**

**Materials**

4-arm-PEG-NH_2_ (*M*_n_ = 10 kDa) was procured from Shanghai Peng Sheng Biotechnology (Shanghai, China). Using a one-pot and two-step technique, γ-selenobutylacetone (γ-SBL) was synthesized from 4-chlorobutyryl chloride and newly prepared NaSeH.^[15]^ Dopamine hydrochloride, polyvinylpyrrolidone (PVP, K-30, *M*_n_ = 40 kDa), ferric chloride and K_4_[Fe(CN)_6_] were obtained from Aladdin Biochemical Technology (Shanghai, China). Solarbio (Beijing, China) supplied Triton X-100. Carbinol was purchased from Concord Technology (Tianjin, China). Beyotime (Shanghai, China) provided the Quantitative Peroxide Assay Kit and 2',7’-dichlorodihydrofluorescein diacetate (DCFH-DA). Abcam (Cambridge, UK) supplied antibodies against F4/80, alpha-smooth muscle actin (α-SMA) and CD31, while Gibco (Grand Island, NY, USA) supplied penicillin‒streptomycin, fetal bovine serum (FBS), and Dulbecco's modified Eagle’s medium (DMEM). The SOD assay kit was purchased from Nanjing Jiancheng Bioengineering Institute (Nanjing, China). NIH-3T3 (a mouse embryonic fibroblast cell line) cells were acquired from the Cell Bank of the Chinese Academy of Sciences (Shanghai, China).

**Polydopamine nanoparticle (PDANP) synthesis**

Two milliliters of ammonia water (25%) was mixed with 80 mL of absolute ethanol and 180 mL of deionized water and stirred at 25°C. Dopamine hydrochloride (1.90 g, 10 mmol) was dissolved in 20 mL of deionized water and poured into the prepared mixture. Finally, the mixture was stirred for 30 h for polymerization at 25°C. Finally, the resultant mixture was washed with deionized water *via* centrifugation at 15,000 rpm for 10 min to obtain PDANPs. The morphology of PDANPs was examined *via* TEM (HT7700, Hitachi, Tokyo, Japan). The particle size and polydispersity index (PDI) of PDANPs were measured *via* dynamic light scattering (DLS) (Zetasizer, Malvern, UK).

**Preparation of PB nanozymes**

Briefly, iron chloride (0.16 g, 1 mmol) and PVP (2.22 g, 20 mmol in terms of monomer) were added to 80 mL of deionized water to make solution A. Solution B was prepared by adding 1 mmol K_4_[Fe(CN)_6_] to 20 mL of deionized water. Solution A was mixed with solution B and vigorously stirred at 60°C for 30 min. The mixture was cooled to 25°C, and PB nanozymes were collected *via* centrifugation at 15,000 rpm for 60 min. The morphology of PB nanozymes was examined *via* TEM. The particle size and PDI of PB nanozymes were measured *via* DLS.

**Synthesis of 4-arm-PEG-SeH** **and hydrogels**

One gram of 4-arm-PEG-NH_2_ (10 kDa) was added to 9 mL of deionized water. Thereafter, SBL was added to the solution at a molar ratio of 4:1 (SBL to 4-arm-PEG-NH_2_), followed by continuous stirring at 25°C for 8 h, resulting in the successful synthesis of 4-arm-PEG-SeH. The solution was placed in air for oxidative crosslinking for 24 h to prepare PEG-Se_2_ hydrogels. Furthermore, PEG-Se_2_ hydrogels loaded with PDANPs and PB nanozymes (DSeP@PB) were prepared. Different amounts of PDANPs and PB nanozymes were added to the 4-arm-PEG-SeH solution to synthesize a series of hydrogels as follows: 4-arm-PEG-SeH (4-arm polyethylene glycol with selenol), PEG-Se_2_ (diselenide-containing polyethylene glycol hydrogels), DSeP (PEG-Se_2_ hydrogels loaded with PDANPs) and DSeP@PB (PEG-Se_2_ hydrogels loaded with PDANPs and PB nanozymes).

**NMR, FTIR, XPS and FESEM analysis**

^1^H NMR spectra were recorded in D_2_O on a Bruker Avance 300 system (Bruker, CA, USA) at 300 MHz. Chemical shifts (δ) were expressed as parts per million relative to D_2_O (4.70 ppm in ^1^H NMR spectra). The reaction between SBL and 4-arm-PEG-NH_2_ was tracked by monitoring the integration of the characteristic peaks of SBL. For Fourier transform infrared spectroscopy (FTIR), 4-arm-PEG-NH_2_ and freeze-dried hydrogels were ground to powder and analyzed using KBr pellets on a Bruker TENSOR 27 FTIR system (Bruker Daltonics Inc., CA, USA). The element composition and valency changes in the polymers were measured *via* X-ray photoelectron spectroscopy (XPS) (Thermo Fisher Scientific, ESCALAB 250 XI, Al KR source, MA, USA). The morphology of the hydrogels was examined on a field emission scanning electron microscope (FESEM, Hitachi S-4700, Tokyo, Japan, 15 kV) after all freeze-dried hydrogel samples were sprayed with a thin gold layer.

**Rheological measurements**

All rheological measurements were performed at 37°C to simulate the human environment. The distance between the rotor and the sample stage was 1 mm when using a 25-mm parallel plate for measurement and testing. The strain sweep settings were as follows: frequency, 1 Hz; strain sweep, 1–1000%. The frequency sweep settings were as follows: strain, 1%; frequency sweep, 1–100 Hz. The time sweep test was performed at a frequency of 1 Hz, strain of 1% and sweep time of 300 s. In addition, the self-healing performance of the hydrogels was tested *via* rheological analysis. The hydrogels were scanned for 120 s alternately under strain conditions of 0.1% and 1000%, with a total of 4 cycles. After a cycle was completed, another cycle of 120 s was run. For the low-shear measurement at 0.1% strain, the frequency remained constant at 10 Hz during the entire process.

**Analysis of tissue adhesion strength**

Fresh porcine skin was cut into rectangular sections of 1 × 3 cm with a thickness of 5 mm and kept moist at all times. First, 0.2 g of the hydrogel sample was used to uniformly coat an area of 1 × 1 cm on the first strip. Subsequently, the second strip was brought into contact with the first strip (the overlap area corresponding to the hydrogel-coated surface), and the resulting lap-shear sample was placed in a humid chamber at 37°C for 1 h before testing. Subsequently, the samples were tested with a cross-head speed of 5 mm/min (the tensile machine was equipped with a 5000-N load cell), and the maximum adhesive strength was determined.

**Hydrogen peroxide degradation assay**

PB nanozymes at different concentrations were mixed with H_2_O_2_ (200 μM) and incubated at 37°C with shaking (100 rpm). At certain time intervals, 20 μL of the solution was removed from the reaction mixture, and the concentration of the remaining H_2_O_2_ was measured *via* a xylenol orange assay according to the manufacturer’s instructions (Quantitative Peroxide Assay Kit, Beyotime, Shanghai, China). Briefly, 50 μL of the sample solution was mixed with 100 μL of the xylenol orange reagent and incubated at 25°C for 30 min. Subsequently, absorbance was measured at 560 nm using a microplate reader (SpectraMax M3, Molecular Devices, California, USA). The absorbance value was converted to the H_2_O_2_ concentration based on a standard curve plotted using known concentrations of pure H_2_O_2_.

**Superoxide scavenging activity**

The superoxide anions generated in the xanthine/xanthine oxidase reaction system were reacted with water-soluble tetrazolium-8 (WST-8) to produce a yellow formazan product. The absorbance of this product was measured at 450 nm using a microplate reader (SpectraMax M3, Molecular Devices, California, USA). The change in absorbance was proportional to the superoxide concentration.

Superoxide scavenging activity (%) = 100 × (A_control_ - A_sample_)/A_control_

In the abovementioned formula, A_control_ and A_sample_ are the absorbance values of the control and sample groups, respectively.

**Biocompatibility assay**

Cell viability was evaluated via MTT assay. Briefly, NIH-3T3 cells were seeded in a 96-well plate (5 × 10^3^ cells/well). Following cell attachment, the hydrogels (0–12.5 mg·mL^-1^) and PB nanozymes (0–200 μg·mL^-1^) were added to the cells. The RL-treated groups were illuminated with RL (730 nm, 1 W/cm^2^, 15 min, VCLHLGD0025017, Blueprint, Beijing, China). After 24 h of incubation, the cells were washed and incubated for 4 h at 37°C in fresh medium containing MTT reagent. Finally, the absorbance of formazan crystals dissolved in DMSO was measured at 490 nm using a microplate reader (SpectraMax M3, Molecular Devices, California, USA).

**In vivo diabetic wound healing evaluation**

Tissue samples were harvested on days 7 and 14, fixed in 4% PFA and embedded in paraffin. Thereafter, 5-µm-thick sections were cut from the paraffin sample and mounted on slides for staining. The samples were stained with H&E and Masson’s trichrome stains for histological analysis according to the manufacturer’s instructions. The slides were observed under a Nikon FHEPSE 80i (Nikon, Japan) microscope.

Tissue samples were harvested on days 7 and 14, embedded in optimal cutting temperature compound (OTC) and stored at -80°C. Thereafter, 5-µm-thick frozen sections were mounted on slides for staining. Immunostaining for α-SMA, CD31, F4/80 and TNF-α was performed to evaluate blood vessel formation and anti-inflammatory effects during wound healing. Slides were immersed in carbinol for 10 min. The samples were permeabilized with 0.5% Triton X-100 and incubated with goat serum at room temperature for 1 h to block nonspecific binding. Samples were then treated with primary antibodies overnight at 4°C. Probed slides were then washed and reacted with fluorophore-conjugated secondary antibodies (ZF-0316, GSZB-BIO, Beijing, China). Finally, the slides were counterstained with the nuclear stain DAPI and mounted using Vectashield mounting solution (Vector Laboratories, Burlingame, CA, USA). Stained samples were observed using fluorescence microscopy (FV1000, Olympus, Tokyo, Japan).


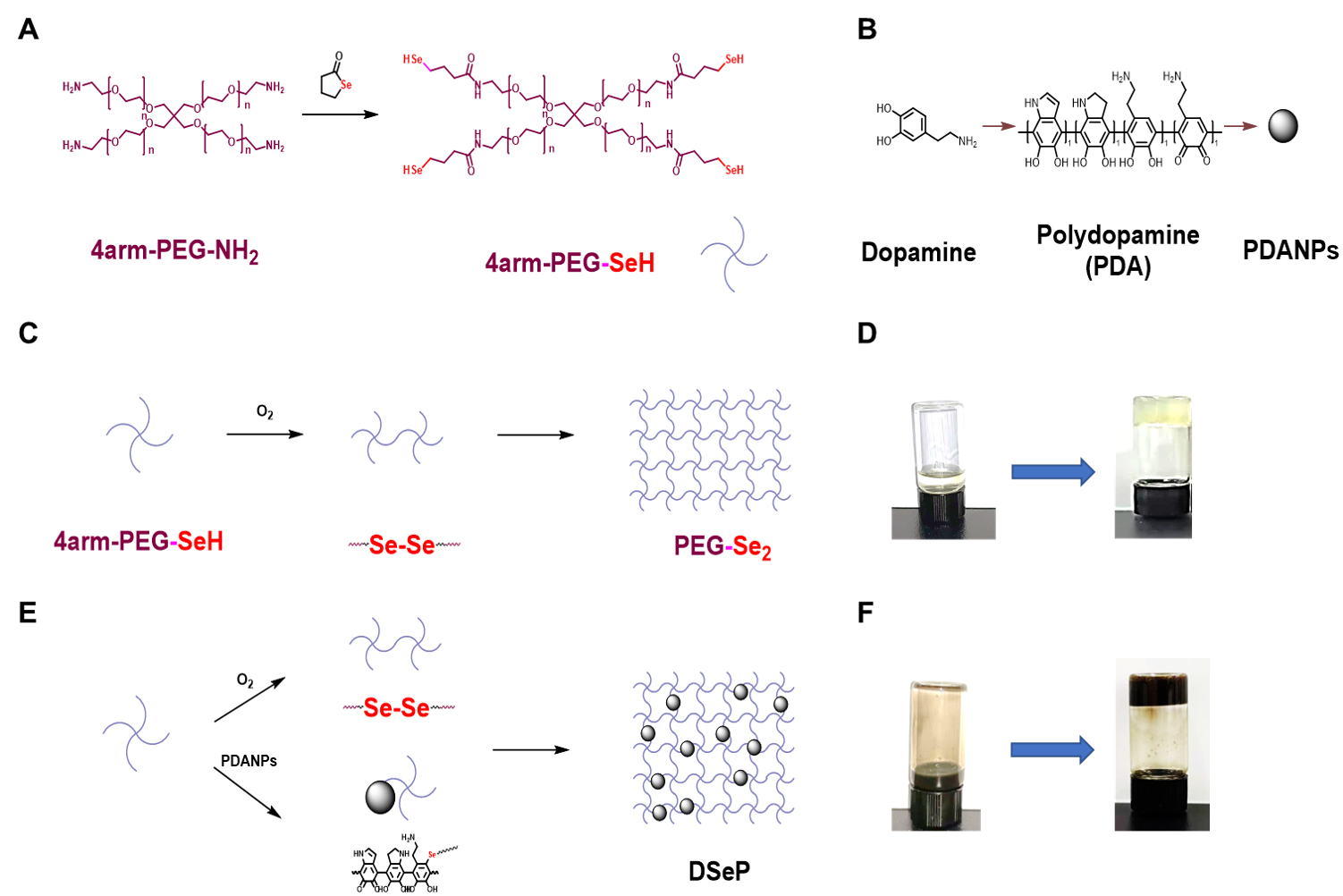


**Fig S1.** Preparation of the 4arm-PEG-SeH and hydrogels. (A) The synthesis of 4arm-PEG-SeH by opening the ring of γ-selenobutylacetone and linking to 4arm-PEG-NH_2_ by the nucleophilic addition reaction. (B) The molecular structure of polydopamine nanoparticles (PDANPs). (C) The preparation scheme of PEG-Se_2_. (D) Photographs of PEG-Se_2_ before and after crosslinking. (E) The preparation scheme of DSeP. (F) Photographs of DSeP before and after cross-linking.


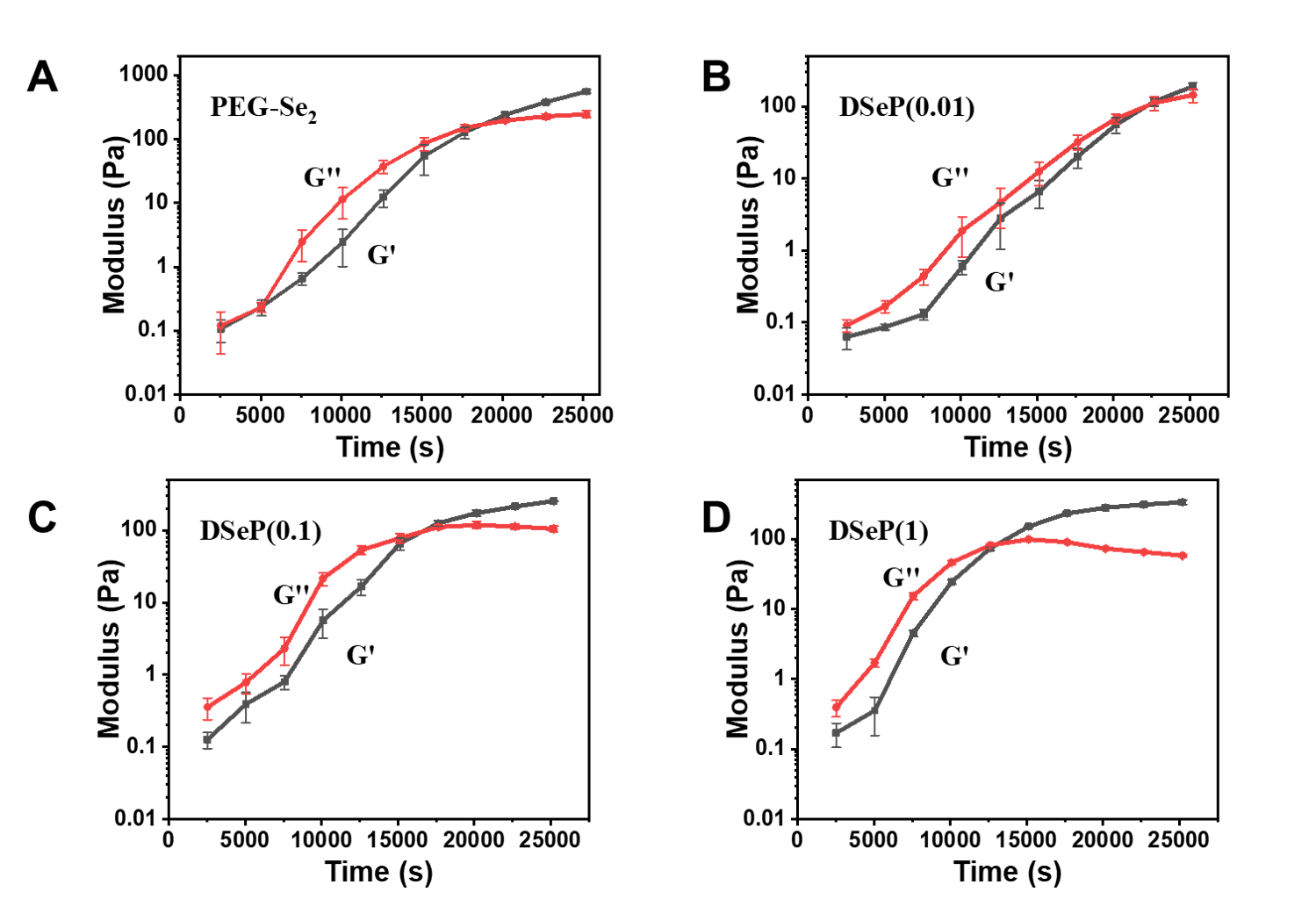


**Fig S2.** Time sweeps for the formation of (A) PEG-Se2, (B) DSeP(0.01), (C) DSeP(0.1), and (D) DSeP(1). Data are presented as the means ± SDs (n = 3).


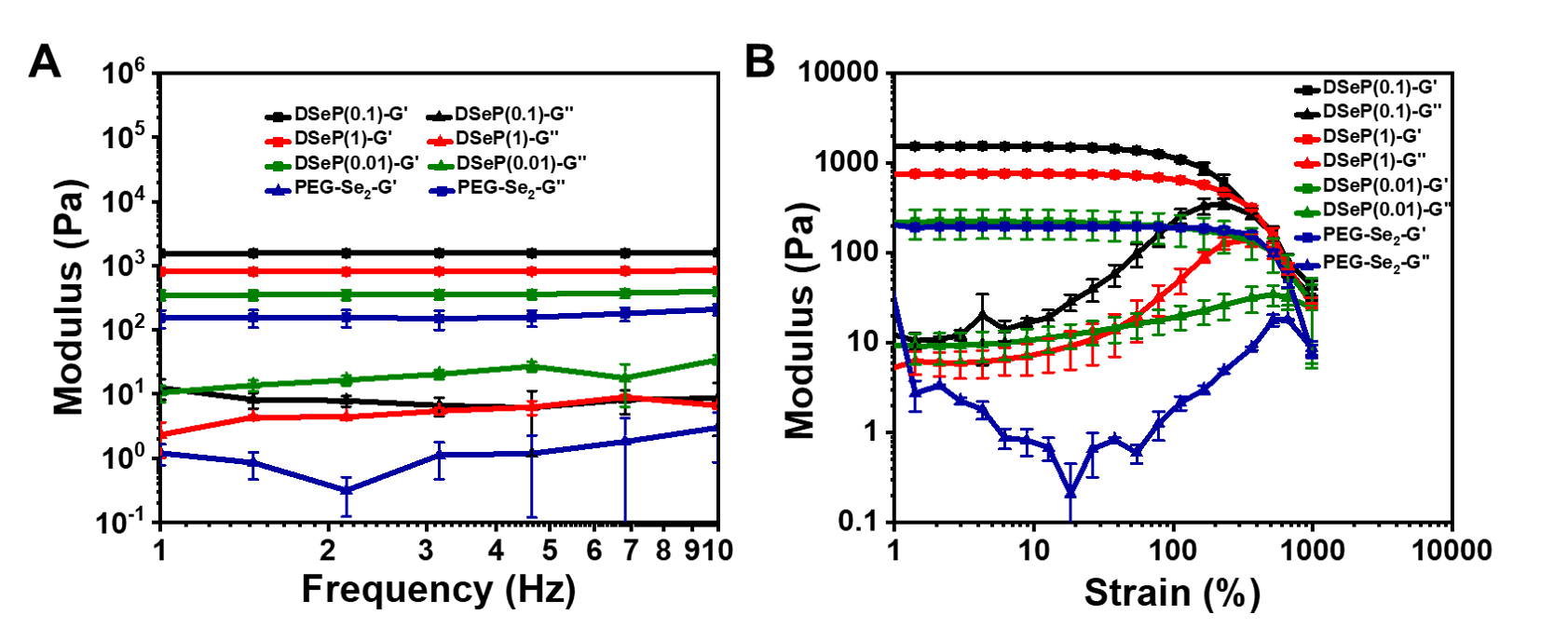


**Fig S3.** Rheological characterization of the hydrogels. (A) Frequency sweep and (B) strain sweep of hydrogels. Data are presented as the means ± SDs (n = 3).


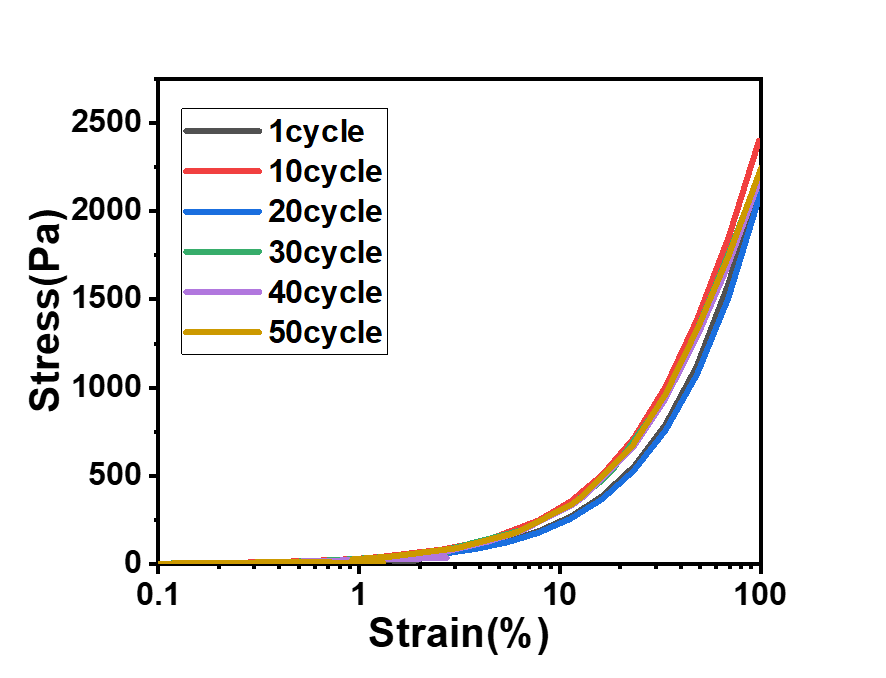


**Fig S4.** The cycling curves of stress–strain for DSeP(0.1).


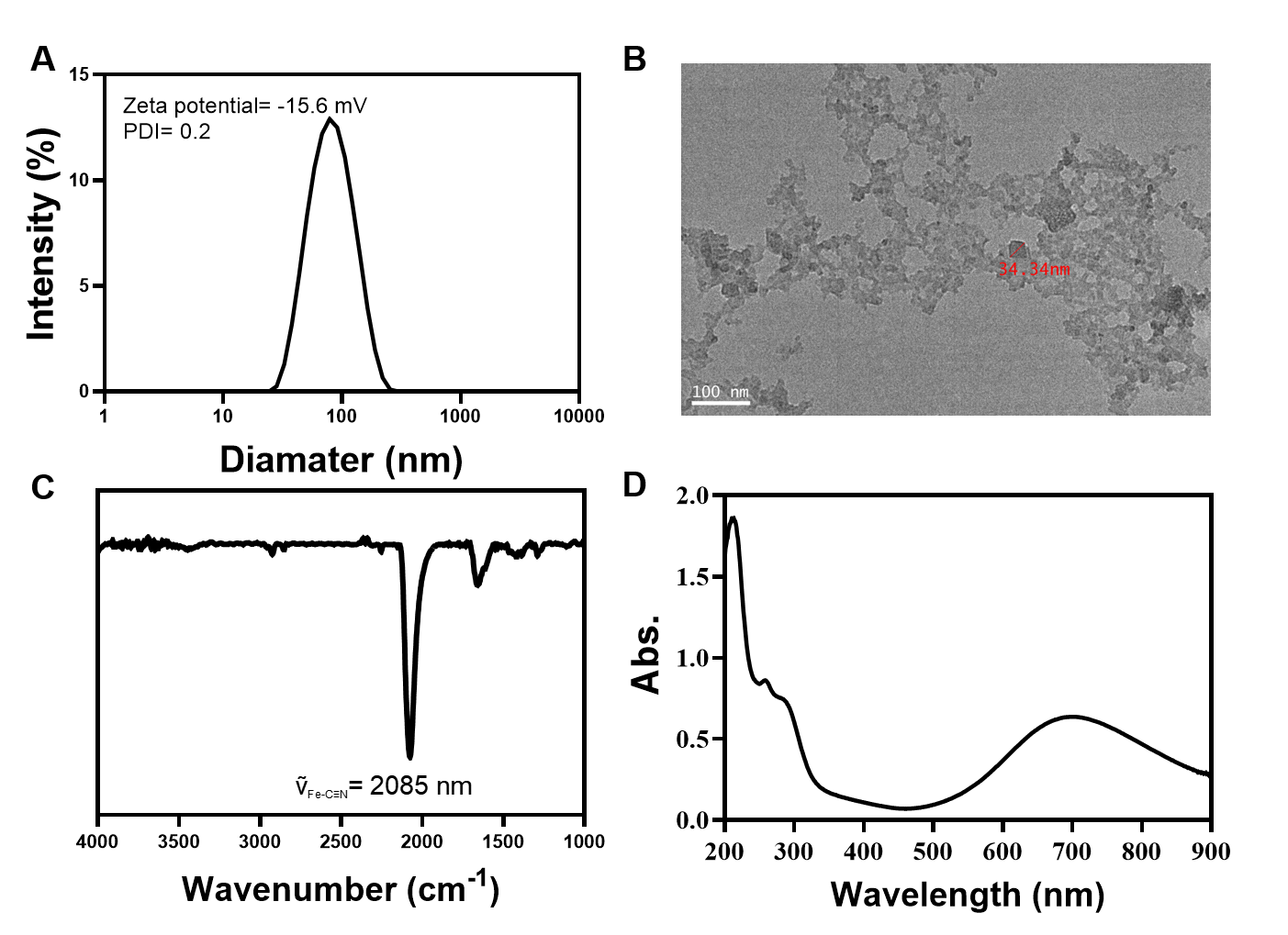


**Fig S5.** The synthesis and characterization of PB nanozymes. (A) Hydrodynamic size distribution of PB nanozymes. (B) TEM image of PB nanozymes. FTIR spectrum (C) and UV–vis absorbance spectrum (D) of PB nanozymes.


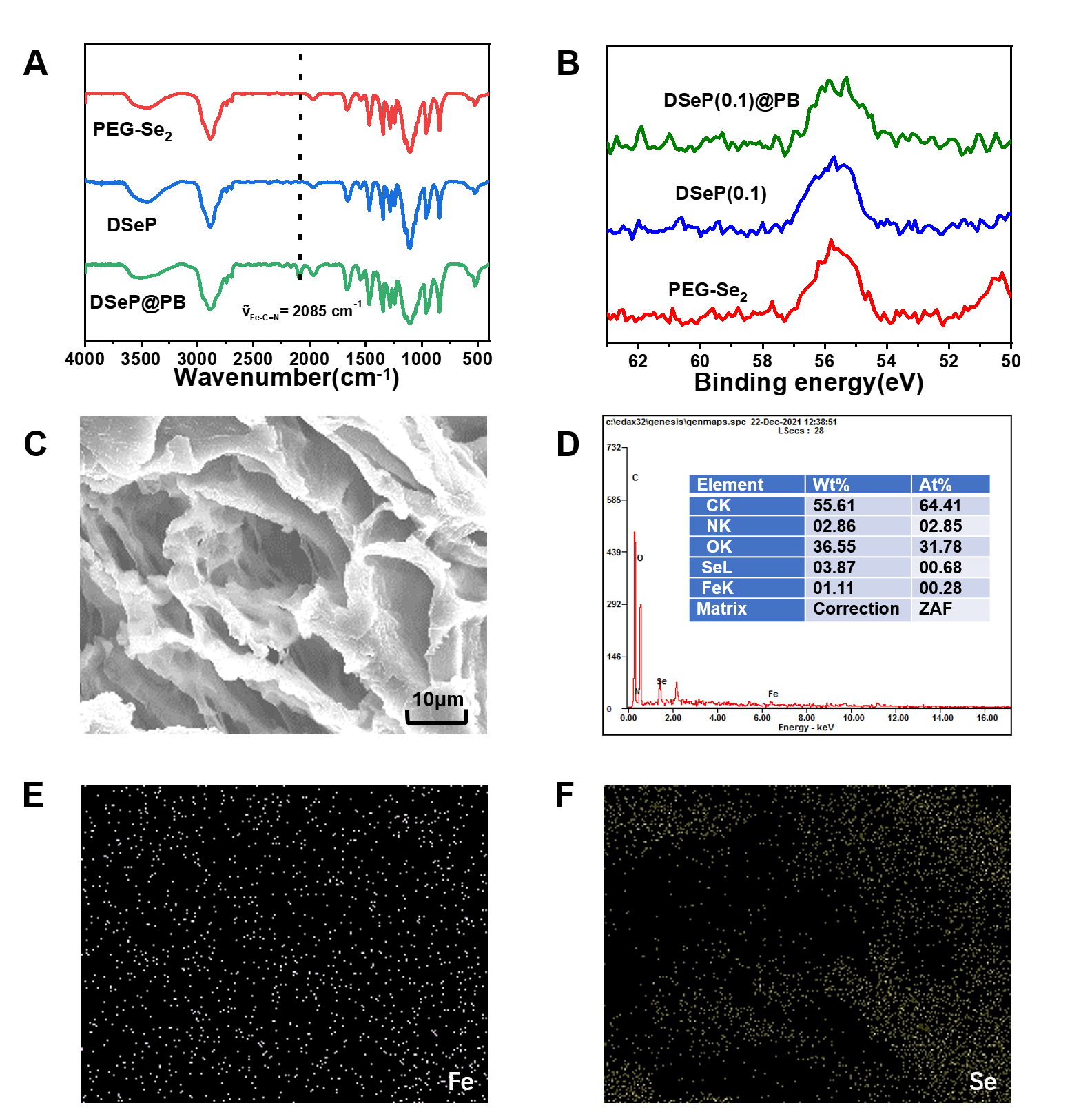


**Fig S6.** Characterization of DSeP@PB. (A) FTIR spectrum of the hydrogels. (B) XPS spectrum of the hydrogels. (C) SEM images of the hydrogels. (D) Elemental content of the hydrogel surface and corresponding elemental mappings of (E) Fe and (F) Se of the hydrogels.


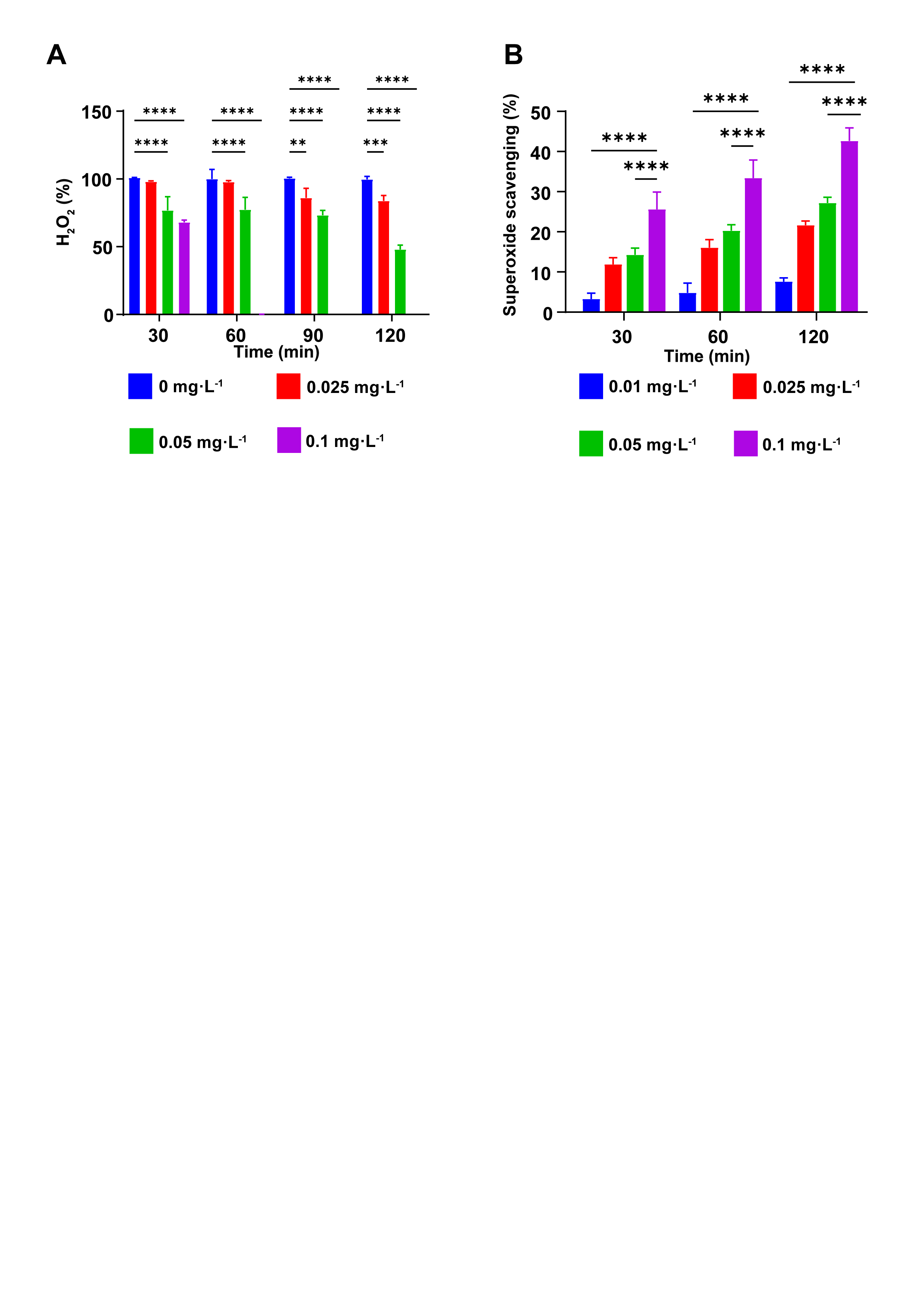


**Fig S7.** In vitro antioxidant assay of PB nanozymes. (A) The ability of PB nanozymes to degrade hydrogen peroxide in a time- and dose-dependent manner. (B) The superoxide scavenging activity of PB nanozymes in a time- and dose-dependent manner. Data are presented as the means ± SDs (n = 3).


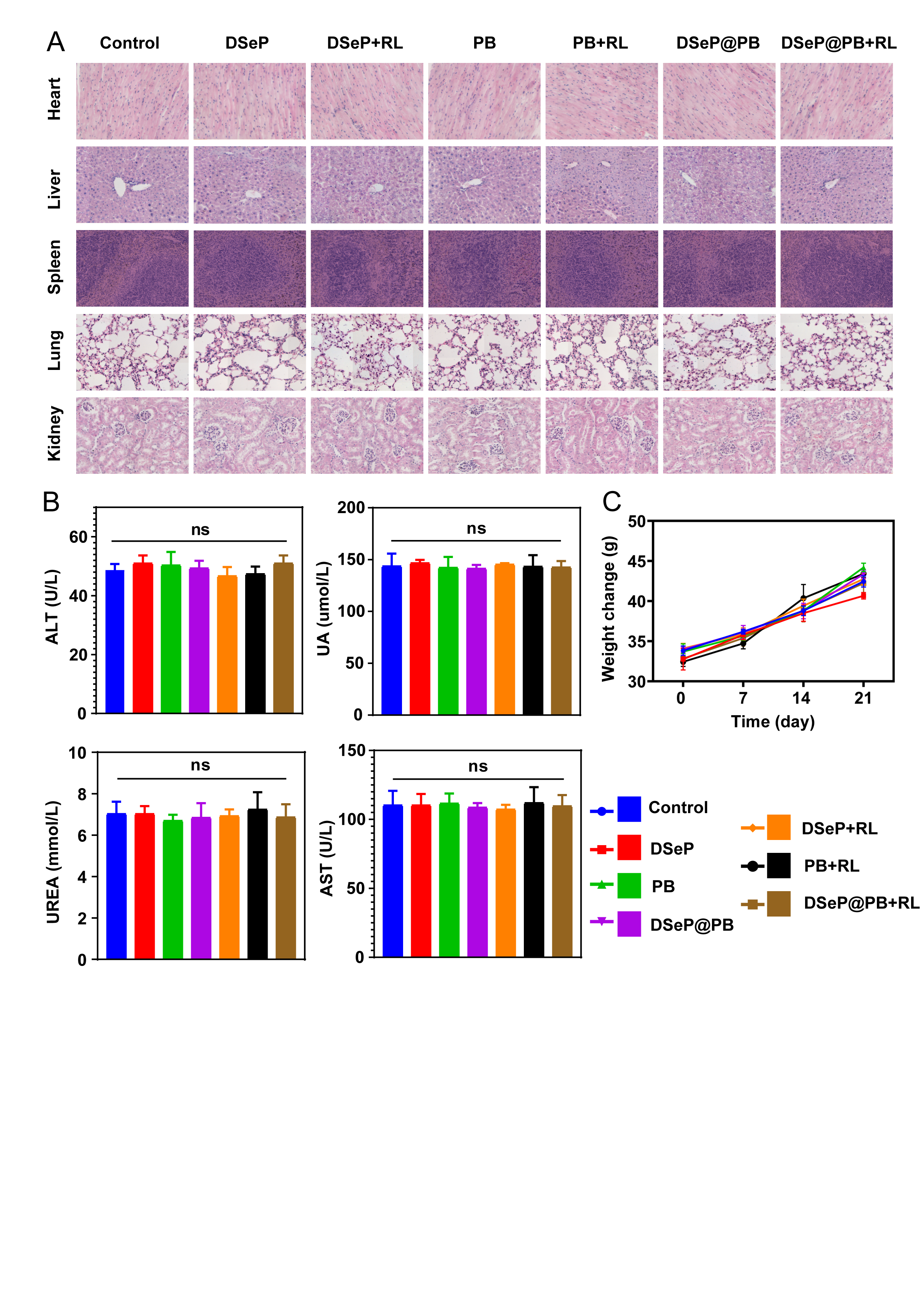


**Fig S8.** Biosafety assay of hydrogels and PB nanozymes. (A) H&E staining of the heart, liver, spleen, lung, and kidney at day 21 after treatment. (B) Liver and kidney function tests of ALT, AST, urea and UA. (C) Weight changes in mice during the treatment period of 21 days. Data are presented as the means ± SDs (n =3).
